# Multi-Targeted Anti-Alzheimer’s Effects of Tri-Sannibat-Phol: Biological Evaluation, Behavioral Validation, and LC-MS/MS Phytochemical Profiling

**DOI:** 10.3390/ph19071063

**Published:** 2026-07-09

**Authors:** Pitchayakarn Takomthong, Pornthip Waiwut, Sumet Kongkiatpaiboon, Khemjira Phemphunananchai, Chantana Boonyarat

**Affiliations:** 1 Faculty of Pharmaceutical Sciences, Khon Kaen University, Khon Kaen 40002, Thailand; ppitcha.t@gmail.com (P.T.); khemjira_ph@kkumail.com (K.P.); 2 Faculty of Pharmaceutical Sciences, Ubon Ratchathani University, Ubon Ratchathani 34190, Thailand; pwaiwut79@yahoo.com; 3 Drug Discovery and Development Center, Office of Advanced Science and Technology, Thammasat University (Rangsit Campus), Pathum Thani 12121, Thailand; sumet_k@tu.ac.th; 4 Center for Research and Development of Herbal Health Products, Khon Kaen University, Khon Kaen 40002, Thailand

**Keywords:** traditional medicine, Tri-sannibat-phol, multi-target therapeutics, *Piper retrofractum*, *Ocimum tenuiflorum* L., *Piper nigrum*

## Abstract

**Background:** Alzheimer’s disease (AD) is a progressive neurodegenerative disorder characterized by oxidative stress, cholinergic dysfunction, and amyloid-β (Aβ) aggregation. Tri-Sannibat-Phol (TSB), a classical Thai polyherbal formulation comprising *Piper retrofractum* fruit, *Ocimum tenuiflorum* root, and *Piper nigrum* root, has been traditionally used for its medicinal properties, yet its anti-AD potential has never been scientifically evaluated. **Methods:** The therapeutic potential of TSB was investigated through in vitro bioassays including antioxidant, acetylcholinesterase (AChE) inhibitory, and anti-Aβ aggregation assays, alongside neuroprotective evaluation in H_2_O_2_-induced SH-SY5Y neuroblastoma cells. Acute oral toxicity was assessed in male ICR mice in accordance with OECD Guideline 420. Cognitive-enhancing effects were evaluated using the modified Y-maze, Novel Object Recognition, and Morris Water Maze tests in a scopolamine-induced amnesic mouse model. LC-MS/MS analysis was performed for phytochemical characterization and chemical standardization of the formulation. **Results:** TSB demonstrated significant antioxidant activity, AChE inhibitory activity, and anti-Aβ aggregation effects, with *P. nigrum* and *O. tenuiflorum* identified as the primary contributing components. Neuroprotective effects were confirmed in H_2_O_2_-induced SH-SY5Y cells, where TSB significantly improved cell viability across concentrations of 1–100 µg/mL. Acute oral toxicity assessment revealed an LD_50_ exceeding 2000 mg/kg, indicating a favorable safety profile. In vivo behavioral studies demonstrated that TSB at medium-to-high doses significantly reversed scopolamine-induced cognitive deficits across all three behavioral tests. LC-MS/MS analysis identified thirteen piperidine alkaloids, with piperine as the dominant constituent at 17.61 ± 0.80% *w*/*w*, proposed as the primary bioactive driver and chemical marker for future quality standardization. **Conclusions:** These findings suggest that TSB exerts multi-targeted anti-AD effects through complementary mechanisms, supporting its potential as a traditional medicine-based therapeutic candidate for further preclinical and clinical investigation.

## 1. Introduction

Alzheimer’s disease (AD) is a chronic and progressive neurodegenerative disorder and the leading cause of dementia worldwide, clinically characterized by memory impairment, cognitive dysfunction, and behavioral changes [1,2]. Although the precise etiology of AD remains unclear, key pathological mechanisms have been identified, including amyloid-β (Aβ) aggregation, tau protein abnormalities, cholinergic deficits, and oxidative stress [3]. Despite decades of research, current treatments primarily alleviate symptoms rather than modify disease progression, underscoring the urgent need for innovative therapeutic strategies capable of targeting the multifactorial nature of AD [4,5].

Natural products and traditional medicinal systems have attracted increasing interest as sources of multi-targeted anti-AD candidates, owing to their structural diversity and broad spectrum of biological activities [6]. Traditional Thai Medicine (TTM), a comprehensive healthcare system practiced in Thailand for centuries and influenced by Ayurvedic principles, Traditional Chinese Medicine, and local traditions, places herbal medicine at its core [7,8]. Many TTM remedies are polyherbal in nature, where the synergistic combination of multiple herbs is believed to enhance therapeutic outcomes and minimize adverse effects [9,10]. This polyherbal philosophy is particularly relevant to AD, where single-target approaches have largely failed, and multi-component formulations offer the potential to simultaneously address several pathological pathways.

Tri-Sannibat-Phol (TSB) is a classical Thai polyherbal formulation officially recognized under the Notification of the Ministry of Public Health No. 3, B.E. 2566, with its traditional use documented in the Compendium of Medical Aid, Volume 1 (B.E. 2494), and the Traditional Thai Medical Treatise of Khun Sophit Bannalak, Volume 1 (B.E. 2513).

In TTM, TSB is conventionally administered orally, most commonly as powdered herbal preparations, capsules, or decoctions, depending on clinical practice. For decoction, equal proportions of the three herbal ingredients are boiled in water, simmered, filtered, and consumed while warm according to traditional practice. TSB comprises three herbal ingredients mixed in equal proportions (*Piper retrofractum* fruit, *Ocimum tenuiflorum* L. root, and *Piper nigrum* root) and has been traditionally prescribed to reduce high fever, manage circulatory-related disorders, and support overall physiological balance. The phytochemical composition of these three components is particularly relevant to AD pathology. Both *P. retrofractum* and *P. nigrum* are rich in piperidine alkaloids, particularly piperine, which has been reported to exhibit acetylcholinesterase (AChE) inhibitory and neuroprotective activities [11,12]. *O. tenuiflorum* is a source of phenolic compounds such as eugenol, with documented antioxidant and anti-amyloidogenic properties [13,14]. Together, these phytochemical profiles suggest that TSB may exert complementary or synergistic effects across multiple AD-relevant pathological mechanisms. However, while these plants have been studied individually, the classical TSB formulation has never been scientifically evaluated against AD.

The present study therefore provides the first systematic scientific evaluation of TSB against AD through an integrative experimental approach. The anti-AD potential of TSB was assessed via in vitro bioassays targeting key pathological mechanisms, including antioxidant activity, AChE inhibition, and Aβ aggregation inhibition, alongside neuroprotective evaluation in H_2_O_2_-induced SH-SY5Y neuroblastoma cells. Acute oral toxicity was established prior to in vivo studies, in which the cognitive-enhancing effects of TSB were validated using the modified Y-maze, Novel object recognition, and Morris water maze tests in a scopolamine-induced amnesic mouse model. LC-MS/MS analysis was further employed to characterize the major phytochemical constituents of TSB and to identify a chemical marker for future quality standardization of the formulation. These findings offer novel insights that may support the development of evidence-based therapeutics derived from Thai traditional medicine.

## 2. Results

### 2.1. Determination of Total Phenolic and Flavonoid Contents

The total phenolic content (TPC) and total flavonoid content (TFC) were calculated using their respective calibration curves, with correlation coefficients of r^2^ = 0.9994 for TPC and r^2^ = 0.9999 for TFC. As summarized in Table 1, the TPC and TFC of TSB were found to be 59.79 ± 8.05 mg GAE/mg extract and 73.01 ± 12.45 mg QE/mg crude extract, respectively. Among the individual plants, *P. nigrum* and *O. tenuiflorum* exhibited the highest phenolic concentrations, while *P. retrofractum* contained the lowest TPC. Conversely, the flavonoid profile showed a different distribution. *P. nigrum* and *P. retrofractum* possessed higher TFC values compared to *O. tenuiflorum*, which recorded the lowest flavonoid content.

### 2.2. Antioxidant Effects

**Figure 1:**
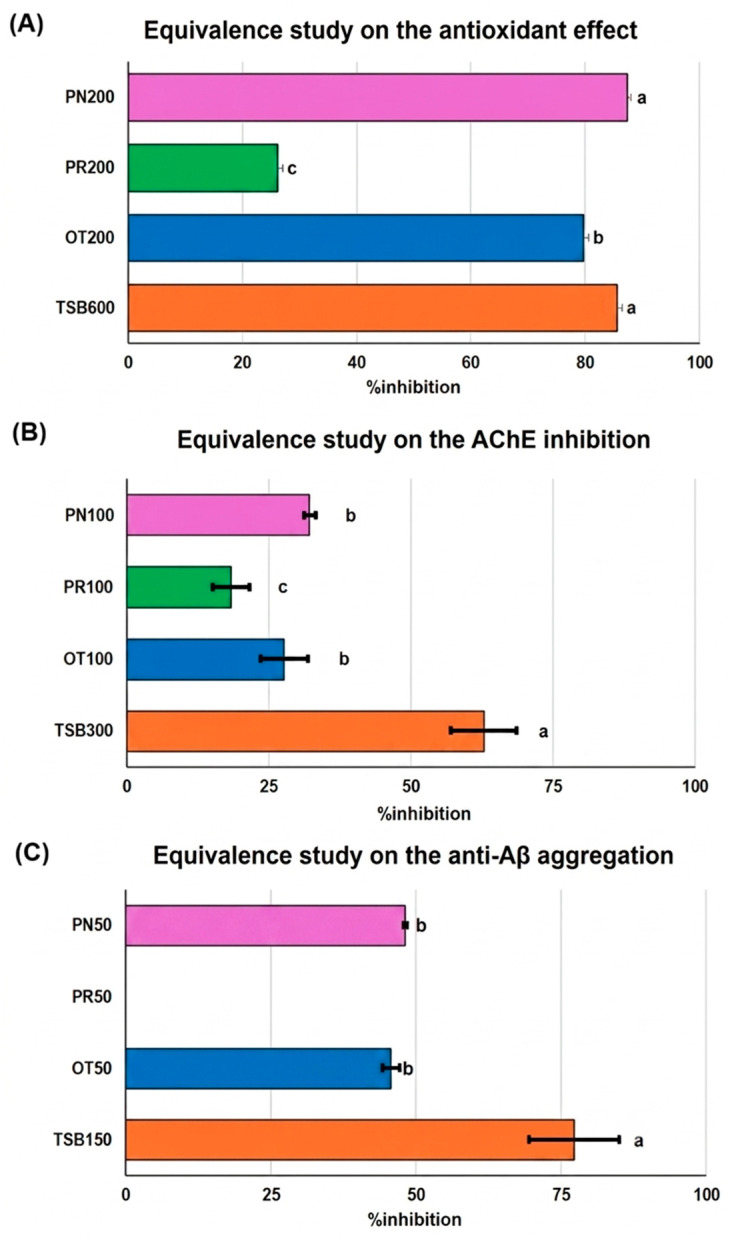
Comparative bioactivity of the TSB formula and its individual herbal components: *O. tenuiflorum* (OT), *P. retrofractum* (PR), and *P. nigrum* (PN). Panels show (**A**) ABTS, (**B**) AChE, and (**C**) Aβ aggregation inhibitory activities. ^a,b,c^ Means in a row with different superscript letters are statistically analyzed by one-way ANOVA (*p* < 0.05).

The antioxidant activity of TSB and individual plants is summarized in Table 2. To elucidate the relative contribution of each component to the overall antioxidant profile, ABTS radical cation scavenging assays were conducted using concentrations proportional to the TSB formulation ratio (Figure 1A). The data demonstrated that *P. nigrum* exhibited the most potent antioxidant activity, and it correlates with its superior TPC. These results suggest that the phenolic constituents within the Piper species are the primary drivers of the electron-transfer-based antioxidant mechanisms in the TSB.

### 2.3. AChE Inhibition

TSB and individual plants demonstrated notable anti-AChE activity in Table 2. To identify the primary drivers of this enzymatic inhibition, individual components were evaluated at concentrations proportional to the TSB formulation (Figure 1B). The results revealed that *O. tenuiflorum* and *P. nigrum* possessed the most potent inhibitory potential. These results indicate that TSB exerts AChE inhibitory effects, likely mediated by its phenolic constituents acting as natural inhibitors to preserve synaptic acetylcholine levels.

### 2.4. Inhibition of Aβ Aggregation

To evaluate the anti-amyloidogenic potential of TSB and its individual components, a thioflavin T assay was performed to assess Aβ aggregation inhibition. Among the tested groups, *O. tenuiflorum* and *P. nigrum* within the TSB demonstrated the strongest inhibitory effects on Aβ aggregation (Table 2 and Figure 1C). In contrast, *P. retrofractum* did not exhibit significant activity. Therefore, the levels of phenolic compounds in *P. nigrum* and *O. tenuiflorum* indicate their potential contribution to their anti-Aβ aggregation effects.

### 2.5. Neuroprotective Effects Against Hydrogen Peroxide (H_2_O_2_)-Induced Cell Damage

**Figure 2:**
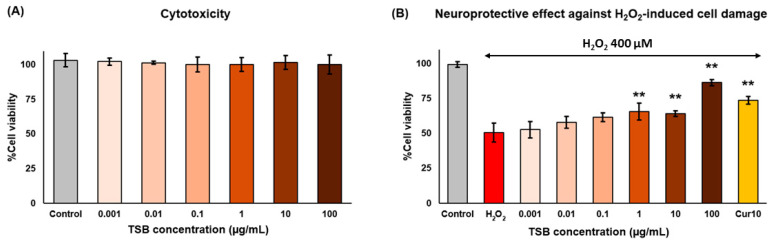
Cytotoxicity (**A**) and neuroprotective effects (**B**) of TSB extract in SH-SY5Y cells. (**A**) Cytotoxicity of TSB following treatment with the indicated concentrations. (**B**) Neuroprotective effects of TSB against H_2_O_2_-induced oxidative damage. SH-SY5Y cells were pretreated with various concentrations of TSB for 2 h, followed by exposure to H_2_O_2_ (400 μM) for 2 h. Curcumin (Cur, 10 μM) was used as the positive control. ** *p* < 0.01 compared to the H_2_O_2_ group, as analyzed by one-way ANOVA.

H_2_O_2_ contributes to oxidative stress by permeating biological membranes and inducing cellular damage. Its accumulation is associated with brain inflammation and neuronal apoptosis, which are commonly observed in neurodegenerative disorders [15,16]. In our study, H_2_O_2_ exposure led to a dose-dependent reduction in the viability of SH-SY5Y cells, consistent with findings from previous research [17]. The cytotoxicity effect of the TSB was investigated to assess its toxicity to cells (Figure 2A). None of the concentrations exhibited toxicity to the cells. TSB was then further evaluated for its neuroprotective effects against H_2_O_2_-induced cell damage. This evaluation aimed to determine its potential in protecting neuronal cells from oxidative stress-related damage. The H_2_O_2_ concentration at 400 µM resulted in 50.50 ± 6.77% cell viability, with curcumin used as the standard. It showed a cell viability of 73.66 ± 2.62%. The TSB results demonstrated that concentrations ranging from 1 to 100 µg/mL could protect cells from oxidative damage (Figure 2B). Therefore, pre-treatment with TSB significantly improved the viability of SH-SY5Y cells exposed to H_2_O_2_ (*p* < 0.01), indicating the neuroprotective effects of TSB in H_2_O_2_-treated SH-SY5Y cells. Despite these protective effects, several limitations should be considered. The H_2_O_2_-induced SH-SY5Y cell model represents an acute oxidative stress condition and primarily reflects antioxidant-associated neuroprotection. Further validation in AD-relevant models, such as Aβ-induced cell toxicity, tau pathology, or neuroinflammatory interactions, will be required to confirm the translational significance of TSB.

### 2.6. In Vivo Studies

#### 2.6.1. Acute Oral Toxicity Study (Sightseeing and Main Study)

The sighting study confirmed the safety of TSB across a wide range of oral doses (5–2000 mg/kg). All treated animals survived the dosing and observation periods, maintaining normal behavior and physiological health until day 14. There were no observable signs of acute toxicity or behavioral distress in any of the dose groups. These results indicate that TSB does not induce acute lethal toxicity at doses up to 2000 mg/kg (Table 3). Following the sighting study, a main study was conducted at the 2000 mg/kg dose level and administered to four additional animals. Consistent with the preliminary findings, all animals survived the 14-day observation period without manifesting any signs of severe clinical toxicity or behavioral distress. Throughout both the sighting and main studies, the body weight of ICR mice treated with TSB remained within normal physiological ranges. All experimental animals demonstrated consistent weight gain over the 14-day observation period (Table 3). Specifically, the mean weekly body weight gain ranged from 27 to 30 g, indicating that the extract did not induce any metabolic suppression or appetite loss at the tested doses. Based on these results, the acute oral toxicity for TSB in male ICR mice is estimated to exceed 2000 mg/kg, indicating a high safety margin for the extract.

#### 2.6.2. Locomotion Activity

**Figure 3:**
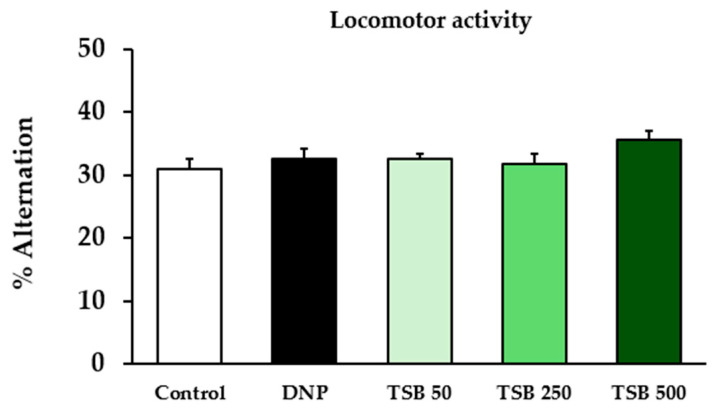
Effect of TSB extract on locomotor activity in the Y-maze, measured by total number of arm entries. Data are presented as mean ± SEM (*n* = 8). Donepezil (DNP) at the dose of 3 mg/kg was used as a reference standard.

Evaluation of locomotor activity is a fundamental control in behavioral studies involving mice models. This assessment determines whether observed shifts in cognitive performance are truly reflective of learning and memory or are merely artifacts of altered motor function. Furthermore, monitoring general activity helps identify potential sedative or stimulant side effects of the experimental compounds, which could otherwise confound the interpretation of cognitive assays. In the present study, Y-maze locomotor assessments confirmed that the treatments did not adversely affect physical movement, as no differences were observed across any of the experimental groups (Figure 3).

#### 2.6.3. Modified Y-Maze

**Figure 4:**
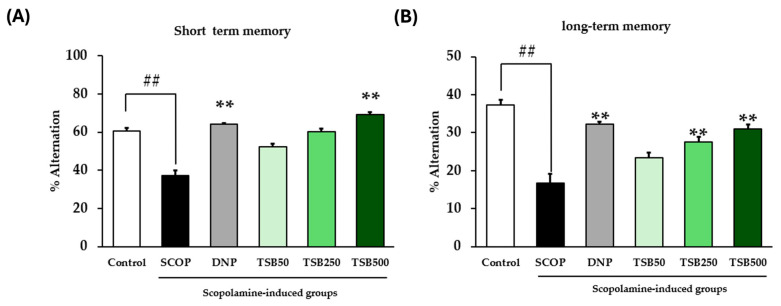
Effect of TSB on short-term (**A**) and long-term (**B**) spatial memory in the modified Y-maze test using a scopolamine-induced amnesia mouse model. Amnesia was induced by intraperitoneal administration of scopolamine (SCOP, 1 mg/kg). Donepezil (DNP, 3 mg/kg) was used as the positive control. Data are presented as mean ± SEM (*n* = 8). ** *p* < 0.01 vs. SCOP group; ^##^ *p* < 0.01 vs. normal control group.

To evaluate the impact of TSB on spatial memory deficits, we utilized the modified Y-maze to assess memory retention via delayed spontaneous alternation [18]. Our study demonstrated that scopolamine (1 mg/kg) induced robust spatial memory deficits, characterized by reduced spontaneous alternation and decreased exploration of unfamiliar arms. Interestingly, the administration of TSB at a high dose (500 mg/kg) significantly reversed these deficits (*p* < 0.01). The improvement in unfamiliar arm exploration suggests that TSB effectively ameliorated both short-term and long-term spatial memory impairments (Figure 4A,B).

#### 2.6.4. Novel Objective Recognition

**Figure 5:**
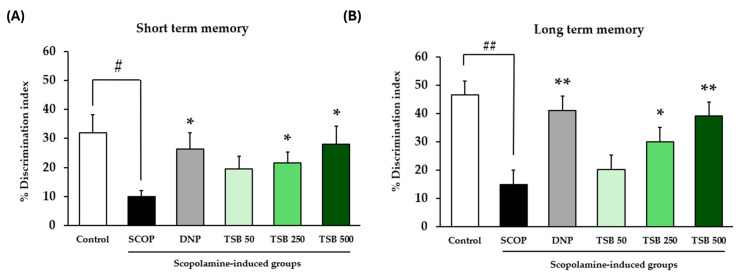
Effect of TSB on non-spatial memory in the novel objective recognition test: (**A**) 5 min delay test (short-term memory) and (**B**) 24 h delay test (long-term memory). Memory impairment was induced by intraperitoneal administration of scopolamine (SCOP, 1 mg/kg). Donepezil (DNP, 3 mg/kg) was used as the positive control. Data are presented as mean ± SEM (*n* = 8). * *p* < 0.05, ** *p* < 0.01 vs. SCOP group; *^#^ p* < 0.05, ^##^ *p* < 0.01 vs. normal control group.

To assess non-spatial recognition memory, we employed the novel objective recognition (NOR) test to determine how effectively mice differentiated between familiar and novel objects [19]. Unlike the Y-maze, which relies on spatial cues, the NOR test evaluates the animal’s innate preference for novelty. Our results showed that scopolamine significantly reduced the time mice spent exploring the novel object, leading to a lower discrimination index (DI) (*p* < 0.05). This suggests that scopolamine-induced cholinergic blockade disrupts the formation and retrieval of object-identity memory. Conversely, both donepezil and medium-to-high doses of TSB significantly elevated the DI in both short- and long-term memory (Figure 5A,B). These improvements suggest that TSB’s bioactive compounds might enhance synaptic plasticity or protect cholinergic neurons against the neurotoxic effects of scopolamine.

#### 2.6.5. Morris Water Maze

**Figure 6:**
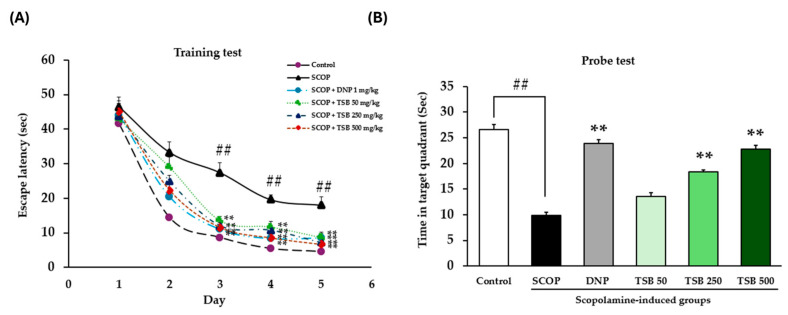
Effect of TSB on spatial learning and memory in the Morris water maze. (**A**) Escape latency during the acquisition phase (days 1–5). (**B**) Time spent in the target quadrant during the probe test on day 6. Memory impairment was induced by intraperitoneal administration of scopolamine (SCOP, 1 mg/kg). Donepezil (DNP, 3 mg/kg) was used as the positive control. Data are presented as mean ± SEM (*n* = 8). ** *p* < 0.01 vs. SCOP group; ^##^ *p* < 0.01 vs. normal control group.

Spatial learning and memory were evaluated using the Morris water maze by measuring escape latency during the acquisition phase and time spent in the target quadrant during the probe test, which reflects the ability to learn and retain the location of a hidden platform, respectively [20,21]. During the acquisition phase, the scopolamine group showed significantly prolonged escape latency compared to the normal control from day 2 onward (Figure 6A, *p* < 0.01), confirming successful induction of spatial memory impairment. Although escape latency gradually declined across all groups throughout the training period, TSB-treated animals at all dose levels reached significance compared to the scopolamine group by day 3 (*p* < 0.01), indicating that TSB facilitated spatial learning recovery in a time-dependent manner. On day 6, the probe test was conducted to assess memory retention in the absence of the platform (Figure 6B). The scopolamine group spent significantly less time in the target quadrant relative to the normal control, reflecting impaired retention of platform location. In contrast, TSB at medium and high doses significantly increased time spent in the target quadrant compared to the scopolamine group (*p* < 0.01), demonstrating that TSB effectively reversed scopolamine-induced spatial memory deficits in a dose-dependent manner.

### 2.7. LC-MS/MS Profiling and Determination of Piperine Content

**Figure 7:**
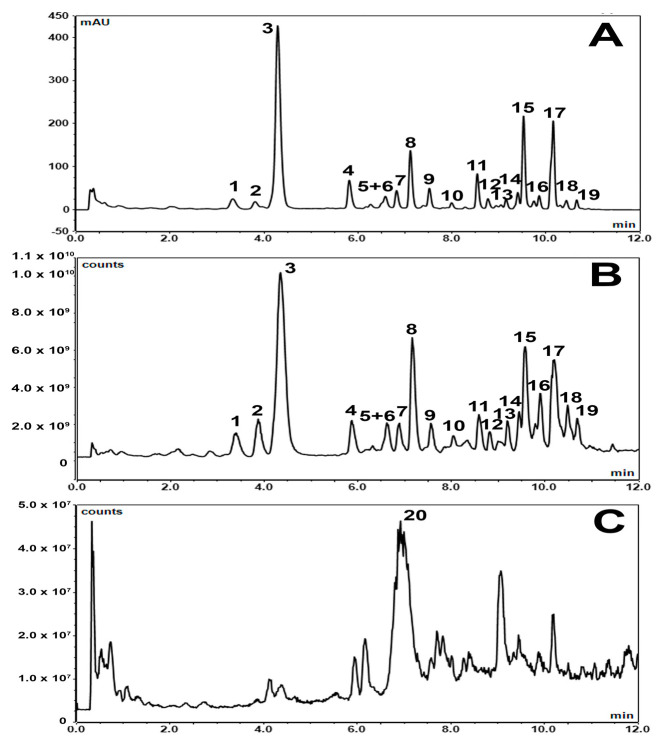
UHPLC chromatographic profile of the Tri-Sannibat-Phol extract (2 mg/mL) detected with (**A**) 254 nm, (**B**) full scan with positive mode at 100–1000 *m*/*z*, and (**C**) full scan with negative mode at 100–1000 *m*/*z*.

TSB has been analyzed using both qualitative and quantitative analyses using UHPLC-DAD-MS/MS. The representative chromatogram and peak area ratio were shown in Figure 7 and Figure S1 and Table S1. Mass spectral data were illustrated in Supplementary Table S2 and Figures S2–S20. The compounds were identified or tentatively identified using mass spectral data compared with NIST, mzCould database, or those reported in the studies. Major constituents in TSB extract were piperine, accompanied by piperidine alkaloids. Piperine could explicitly be identified from [M + H]^+^ ion at *m*/*z* 286.1434 (calculated 286.14377). The fragment mass of piperine at *m*/*z* 201 indicated the cleavage of the carboxyamide moiety as previously described [22]. For the quantitative analysis, piperine content was used as a chemical marker for TSB. The quantification of piperine was done using the data obtained from DAD detected at UV 337 nm. A calibration curve was constructed with peak area versus the concentration of standard using the least squares method as shown in Figure S1. Piperine showed a good linear relationship in the range of 3.9–500 μg/mL with a coefficient of determination (r^2^) of 0.9993 (Equation: Y = 0.5015X − 0.3614). The concentration of piperine in TSB was 17.61 ± 0.80% *w*/*w*.

## 3. Discussion

This study explored the therapeutic potential of TSB, a classical Thai polyherbal formulation, against AD through a combination of in vitro bioassays, acute oral toxicity assessment, behavioral animal models, and LC-MS/MS phytochemical characterization. TSB consists of three herbal components (*Piper retrofractum* fruit, *Ocimum tenuiflorum* root, and *Piper nigrum* root), and each is individually reported to possess neuroprotective and antioxidant properties, yet previously unexamined as a combined formulation related to AD. Despite the well-documented pharmacological activities of its individual constituents, the therapeutic potential of TSB as an integrated polyherbal preparation had never been systematically assessed, representing a significant gap in the scientific validation of Thai traditional medicine. The present study therefore represents the first systematic scientific evaluation of TSB targeting the core pathological mechanisms of AD, including oxidative stress, cholinergic dysfunction, and amyloid-β aggregation, and cognitive impairment, while also establishing the safety profile and chemical standardization basis of the formulation for future development.

Oxidative stress, cholinergic dysfunction, and Aβ aggregation are among the most extensively investigated pathological mechanisms in AD, and natural product-derived formulations capable of addressing these targets simultaneously have attracted considerable interest as multi-targeted therapeutic candidates [4]. The failure of single-target AD therapeutics in clinical trials has reinforced the rationale for multi-component herbal formulations, which may confer complementary or synergistic benefits across several pathological pathways simultaneously [4,23]. TSB demonstrated meaningful activity across all three in vitro parameters, with particularly notable contributions from *P. nigrum* and *O. tenuiflorum*.

Antioxidants are essential in combating AD by neutralizing free radicals and reducing oxidative stress [24]. Numerous natural antioxidants have demonstrated efficacy in AD models, highlighting their therapeutic potential [25]. Consequently, targeting reactive oxygen species through natural antioxidant compounds presents a promising strategy for anti-AD drug development. The antioxidant activity of TSB was primarily driven by *P. nigrum*, which exhibited the highest total phenolic content among the individual components, consistent with literature linking phenolic density to free radical scavenging capacity [24,26]. The relatively lower TPC of *P. retrofractum* compared with the other two components likely accounts for its modest contribution to the overall antioxidant profile of TSB, suggesting that *P. nigrum* and *O. tenuiflorum* are the principal antioxidant drivers within the formulation. For AChE inhibition, TSB produced an IC_50_ of 242.20 ± 0.87 µg/mL, with both *O. tenuiflorum* and *P. nigrum* identified as primary contributors, supported by previous reports of piperine acting as a competitive AChE inhibitor [12] and significant anti-AChE activity reported for *O. tenuiflorum* [27]. Although the inhibitory potency of TSB was lower than that of donepezil, this is expected for crude herbal extracts and is consistent with the activity range reported for other plant-derived AChE inhibitors. The combined contribution of multiple phenolic and alkaloid constituents within TSB may result in additive or synergistic inhibition that is not fully captured by the IC_50_ of any single component. For anti-Aβ aggregation, *O. tenuiflorum* and *P. nigrum* again exhibited the strongest inhibitory effects, while *P. retrofractum* was inactive, possibly reflecting structural requirements for Aβ fibril interference not met by its alkaloid constituents. The anti-amyloidogenic effects of eugenol from *O. tenuiflorum* [14] and piperine from *P. nigrum* [12] reported in prior studies support the observed activity profile of TSB. These suggest that multiple constituents within TSB may contribute to Aβ aggregation inhibition through distinct structural interactions.

To model oxidative stress-related neuronal damage, we employed the H_2_O_2_-induced SH-SY5Y cell model, which closely mimics the elevated ROS environment in AD-affected brain tissue [15]. TSB showed no cytotoxicity across the tested range and significantly improved cell viability following H_2_O_2_ challenge at concentrations of 1–100 µM, with protection comparable to the curcumin standard (*p* < 0.01). These effects are consistent with the antioxidant activity of TSB constituents, particularly eugenol and phenolics from *P. nigrum*, previously shown to attenuate H_2_O_2_-induced neuronal apoptosis [13,17]. The neuroprotective activity of TSB across a broad concentration range (1–100 µg/mL) suggests that multiple antioxidant constituents within the formulation contribute to sustained protective efficacy. This may explain why TSB maintains activity at concentrations where individual compounds alone might be insufficient. However, it should be noted that this model primarily reflects antioxidant-driven neuroprotection. Validation in Aβ-induced cytotoxicity or neuroinflammatory models would be needed to more fully characterize the neuroprotective scope of TSB.

Evaluation of acute oral toxicity is an essential prerequisite for advancing herbal extracts toward in vivo efficacy studies. In accordance with OECD Guideline 420, all animals survived both the sighting phase (5–2000 mg/kg) and the main study (2000 mg/kg), with no clinical signs of toxicity or behavioral distress observed throughout the 14-day period. Consistent body weight gain across all groups confirmed the absence of metabolic suppression at any tested dose. The acute oral LD_50_ of TSB therefore exceeds 2000 mg/kg, placing it in a low acute hazard category under OECD classification. According to the OECD Guideline 420, substances with an LD_50_ exceeding 2000 mg/kg are considered to have low acute oral toxicity, indicating that TSB poses minimal acute hazard at the doses tested. This favorable safety profile is consistent with the traditional human use of TSB and supports its suitability for further preclinical investigation.

Scopolamine, a non-selective muscarinic receptor antagonist, disrupts hippocampal cholinergic signaling and is among the most established tools for inducing amnesia in rodents [3]. The scopolamine model is particularly well-suited for evaluating the functional consequences of cholinergic deficits, which represent one of the earliest and most consistent features of AD [28], and allows direct pharmacological benchmarking against clinically used AChE inhibitors such as donepezil. Locomotor assessments in the Y-maze confirmed no significant differences in motor activity across groups, ruling out confounding effects on cognitive performance. In the modified Y-maze, TSB at 500 mg/kg significantly restored unfamiliar arm exploration in both short- and long-term trials, reversing scopolamine-induced spatial memory deficits to levels comparable to donepezil (*p* < 0.01). The dose-dependent nature of this effect, with 500 mg/kg producing the most consistent reversal, suggests a threshold-dependent pharmacological response that may reflect concentration-dependent BBB penetration of key alkaloids such as piperine [29]. Also, these results align with the well-established cholinergic hypothesis, where scopolamine acts as a muscarinic receptor antagonist to disrupt hippocampal-dependent learning [30]. In the NOR test, medium-to-high doses of TSB significantly elevated the discrimination index, suggesting cognitive benefits extending beyond hippocampal spatial circuits to perirhinal cortex-dependent recognition memory. The improvement in the NOR discrimination index is particularly noteworthy because perirhinal cortex-dependent recognition memory is among the earliest cognitive domains to deteriorate in AD patients, making the NOR test a translationally relevant indicator of early-stage cognitive protection [31,32]. In the Morris Water Maze, TSB reduced escape latency significantly by day 3 (*p* < 0.01) of acquisition, and medium-to-high doses resulted in greater time spent in the target quadrant during the probe test, indicating improved spatial memory retention consistent with prior reports on piperine-containing extracts [11]. The gradual improvement in escape latency across the acquisition phase is consistent with a learning facilitation mechanism rather than a simple anxiolytic or locomotor confounding, further supporting the conclusion that TSB enhances true spatial learning capacity. Collectively, TSB ameliorated scopolamine-induced deficits across multiple memory domains, supporting a broad cognitive-enhancing profile consistent with multi-target rather than single-mechanism activity.

Furthermore, this study employed male animals only, and future studies incorporating both sexes will be necessary to establish whether the cognitive-enhancing effects of TSB are generalizable across sexes. Sex differences in AD pathology and cholinergic responsiveness are well-documented, with female animals generally showing greater vulnerability to Aβ accumulation and tau pathology, making the inclusion of female subjects an important consideration for future preclinical studies aiming to maximize translational relevance [33,34].

UHPLC-DAD-MS/MS analysis identified thirteen piperidine alkaloids in TSB, with piperine as the dominant constituent at 17.61 ± 0.80% *w*/*w*, consistent with the known alkaloid profile of *P. retrofractum* and *P. nigrum* [26,35]. Piperine was confirmed by its [M + H]^+^ ion at *m*/*z* 286.1434 and diagnostic fragment at *m*/*z* 201, in agreement with reported fragmentation patterns [22]. Its pharmacological significance lies in its high BBB permeability and documented capacity to inhibit AChE, reduce hippocampal oxidative stress, and attenuate Aβ-induced neuronal damage [11,12], making it the most plausible primary bioactive driver of the multi-targeted AD-relevant effects observed in this study. Beyond piperine, the identification of twelve additional piperidine alkaloids in TSB suggests that the overall pharmacological activity of the formulation is unlikely to be attributed to a single compound alone. Minor alkaloids such as those co-eluting with piperine in the chromatographic profile may contribute to the observed biological effects through additive or synergistic interactions, a phenomenon commonly observed in polyherbal preparations where the whole extract outperforms isolated fractions. From a standardization perspective, the high and reproducible piperine content positions it as a robust chemical marker for quality control of future TSB-based preparations. The establishment of piperine as a quantitative marker is a critical step toward the regulatory acceptance and industrial scalability of TSB, as batch-to-batch consistency in marker compound concentration is a prerequisite for pharmaceutical-grade herbal product development under both Thai FDA and international quality standards.

TSB demonstrated multi-targeted activity against AD-relevant mechanisms by modulating oxidative stress, AChE activity, Aβ aggregation, and neuroprotection, resulting in significant cognitive enhancement across various behavioral models. While the scopolamine model is well-validated for evaluating cholinergic-dependent cognition, future studies using transgenic AD models would better establish translational relevance. Subsequent investigations measuring AChE activity and Aβ levels in brain tissue would provide in vivo confirmation of target engagement, and pharmacokinetic profiling of piperine and related alkaloids will be essential to inform optimal dosing for future preclinical and clinical development. Taken together, the converging evidence from in vitro bioassays, cellular neuroprotection studies, acute safety profiling, behavioral validation, and phytochemical characterization collectively positions TSB as a scientifically credible and multi-targeted traditional Thai herbal formulation with promising potential for further development as an AD therapeutic candidate.

## 4. Materials and Methods

### 4.1. Formula Preparation

TSB was prepared as a polyherbal formulation comprising equal proportions of *Piper retrofractum* fruit, *Ocimum tenuiflorum* L. root, and *Piper nigrum* root. All plant materials were obtained from Chao Phya Abhaibhubejhr Hospital, Prachinburi Province, Thailand, and botanical identification was carried out by Benjawan Leenin, Head of the Traditional Knowledge Center, Chao Phya Abhaibhubejhr Hospital Foundation. Voucher specimens were deposited at the hospital’s institutional herbarium under accession numbers ABH-TSB1 (*O. tenuiflorum*), ABH-SSY2 (*P. retrofractum*), and ABH-SSY4 (*P. nigrum*). Prior to extraction, each plant material underwent post-harvest processing, including cleaning, drying in a hot air oven at 50 °C, and grinding with an herbal grinder. The resulting powder was sieved through a 60-mesh analytical sieve and collected for subsequent use. Ethanolic extraction was performed by macerating the dried TSB powder mixture (150 g) and each individual plant powder (50 g) in 95% ethanol at room temperature for seven days, followed by filtration. The filtrates were concentrated under reduced pressure and freeze-dried, yielding extraction percentages of 10.11%, 15.46%, 8.69%, and 8.31% for TSB, *P. retrofractum*, *O. tenuiflorum*, and *P. nigrum*, respectively. All crude extracts were stored at 4 °C until use.

### 4.2. Investigation of Total Phenolic Content and Total Flavonoid Content

TPC was quantified by the Folin–Ciocalteu (FC) colorimetric method [36]. Each extract (10 µL) was reacted with FC reagent (75 µL; Sigma-Aldrich, SM Chemical supplies Co., Ltd., Bangkok, Thailand) for 5 min in the dark, followed by the addition of 7.5% (*w*/*v*) Na_2_CO_3_ (75 µL; Unilab Pharmaceuticals, Bangkok, Thailand) and further incubation for 2 h. Absorbance was recorded at 700 nm, and results were expressed as µg gallic acid equivalents per mg crude extract (µg GAE/mg CE) using a gallic acid calibration curve (Sigma-Aldrich, SM Chemical supplies Co., Ltd., Bangkok, Thailand).

TFC was measured by the AlCl_3_ colorimetric method [36]. Briefly, each extract (20 µL) was combined with 2.5% (*w*/*v*) AlCl_3_ (15 µL), CH_3_COONa solution (100 g/L, 20 µL; both Sigma-Aldrich, SM Chemical supplies Co., Ltd., Bangkok, Thailand), and distilled water (145 µL), and absorbance was read at 450 nm after 15 min. TFC values were expressed as µg quercetin equivalents per mg crude extract (µg QE/mg CE) against a quercetin standard curve (Sigma-Aldrich, SM Chemical supplies Co., Ltd., Bangkok, Thailand).

### 4.3. Investigation of Antioxidant Effect Using 2,2′-azino-bis-(3-ethylbenzothiazoline-6-sulfonic acid) (ABTS) Assay

The ABTS radical cation stock solution was prepared by mixing 7 mM ABTS (Sigma-Aldrich, SM Chemical supplies Co., Ltd., Bangkok, Thailand) with 2.45 mM potassium persulfate (K_2_S_2_O_8_) and allowing the reaction to proceed for 12 h at room temperature in the dark. Prior to use, the stock solution was diluted with ethanol until an absorbance of 0.70 ± 0.02 at 700 nm was achieved, yielding the working solution. For the assay, each extract (50 µL) was combined with the ABTS working solution (100 µL) and incubated for 15 min at room temperature, after which absorbance was recorded at 700 nm [36]. Trolox was included as a positive control, as it is the standard reference compound widely used for benchmarking antioxidant capacity in ABTS-based assays, enabling comparison of results across studies.

### 4.4. Investigation of Acetylcholinesterase Inhibitory Activity

AChE inhibitory activity was assessed using a slightly modified colorimetric method based on Ellman’s procedure [37]. Briefly, each extract (25 µL) was incubated with acetylthiocholine iodide (ATCI, 1 mM, 25 µL), 0.1 M phosphate-buffered saline (PBS, 50 µL), 5,5′-dithiobis-(2-nitrobenzoic acid) (DTNB, 1 mM, 125 µL), and AChE derived from electric eel type VI-S (Sigma-Aldrich, SM Chemical supplies Co., Ltd., Bangkok, Thailand). The enzymatic reaction was monitored spectrophotometrically at 405 nm at 30 s intervals over a 5 min period. Donepezil was used as the positive control, as it is the most widely prescribed cholinesterase inhibitor in current clinical AD management, providing a clinically relevant benchmark for comparison of AChE inhibitory potency.

### 4.5. Investigation of Amyloid Beta Aggregation Inhibition

Inhibition of Aβ aggregation was evaluated using the Thioflavin T (ThT) fluorescence assay [36]. Briefly, each extract (5 µL) was combined with 10 µM Aβ_1−42_ peptide (20 µL) and incubated in the dark for 48 h to allow fibril formation. Following incubation, 5 µM ThT solution (175 µL) prepared in glycine/NaOH buffer (pH 8.5) was added, and fluorescence intensity was measured with excitation at 446 nm and emission at 490 nm. Curcumin was included as a positive control based on its well-documented ability to inhibit Aβ aggregation, having been shown to suppress fibril formation and disaggregate preformed Aβ fibrils, making it a widely accepted reference compound for in vitro anti-amyloidogenic assays [38].

### 4.6. Cytotoxicity and Neuroprotective Effect on Hydrogen Peroxide (H_2_O_2_)-Induced Cell Damage

The human neuroblastoma SH-SY5Y cell line (ATCC, CRL-2266) was maintained in DMEM/Ham’s F-12 medium (Sigma-Aldrich, SM Chemical Supplies Co., Ltd., Bangkok, Thailand) supplemented with 10% fetal bovine serum (FBS) and penicillin-streptomycin (Gibthai, GT Chemical supplies Co., Ltd., Bangkok, Thailand), in a humidified incubator at 37 °C with 5% CO_2_. Cell morphology was routinely monitored throughout the culture period. Prior to treatment, cells were seeded into 96-well plates at a density of 5 × 10^5^ cells/mL and allowed to adhere overnight.

For the cytotoxicity assessment, cells were exposed to TSB or individual plant extracts at various concentrations for 2 h, after which cell viability was determined. To evaluate neuroprotective effects, cells were pre-treated with each extract at various concentrations for 2 h. The culture medium was then removed and replaced with H_2_O_2_ solution to induce oxidative stress-related neuronal damage, followed by an additional 2 h incubation before cell viability assessment. Cell viability was quantified using the MTT assay, with absorbance measured at 550 nm [36]. Curcumin was used as a positive control given its well-established antioxidant and neuroprotective properties in H_2_O_2_-induced SH-SY5Y cell injury models [36,39].

### 4.7. In Vivo Studies

#### 4.7.1. Preparation of Drugs

Scopolamine was prepared in a normal saline solution, while donepezil and TSB were administered in a corn oil vehicle. All dosages were adjusted based on individual mouse body weight. Scopolamine was administered at 1 mg/kg for cognitive impairment induction. Donepezil (Sigma-Aldrich, SM Chemical Supplies Co., Ltd., Bangkok, Thailand) at 3 mg/kg served as a positive control due to its proven efficacy in mitigating scopolamine-induced memory deficit [36].

#### 4.7.2. Acclimatization and Classification of Animals

**Figure 8:**
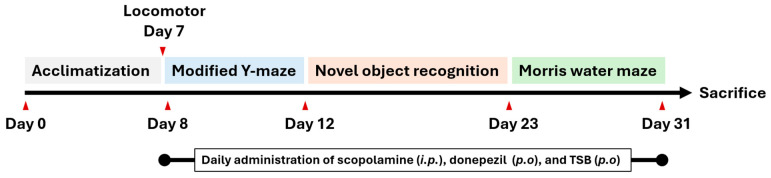
Experimental design and schedule.

The study protocols received approval from the KKU Institutional Animal Care and Use Committee (IACUC-KKU-5/68). Personnel conducted all procedures in strict accordance with Thai IACUC animal welfare guidelines. To prioritize animal well-being, the team implemented a daily monitoring protocol to identify humane endpoints based on health assessments and the mouse grimace scale. Male ICR mice (*n* = 48, 6 weeks old, 34–44 g) from the Northeast Laboratory Animal Center (KKU) were acclimatized for one week with standard housing and free access to food/water. After daily weight monitoring, they were randomly divided into six groups: a corn oil control, a scopolamine-only group, and four treatment groups (donepezil or TSB at 50, 250, or 500 mg/kg) followed by scopolamine. Oral treatments (*p.o.*) were given 1 h before scopolamine (*i.p.*) induction, which occurred 30 min before testing (Figure 8). Mice that failed to respond to environmental challenges or remained immobile were excluded from behavioral assessments. Male ICR mice were used in this study to minimize variability in behavioral performance associated with hormonal fluctuations across the estrous cycle in female animals.

#### 4.7.3. An Acute Toxicity Study

Acute oral toxicity of TSB was evaluated in male ICR mice in accordance with OECD Guideline 420 [40]. The study began with a sighting phase to determine the appropriate starting dose for the main study. Initially, a single animal received a dose of 5 mg/kg, with clinical observations conducted at 0.5, 1, 2, 3, and 4 h post-administration. Following a 24 h survival period, subsequent animals were administered sequentially at 50, 300, and 2000 mg/kg. Upon the survival of the animal at the maximum dose (2000 mg/kg) during the sighting phase, a main study was initiated using four additional animals at that same dose level. All subjects across both phases were monitored daily for 14 days to identify any delayed clinical signs or mortality. Mortality and morbidity were monitored twice daily, focusing on physiological and behavioral indicators including changes in skin, fur, ocular health, and mucous membranes. Specific attention was directed toward detecting signs of autonomic or central nervous system distress, such as tremors, convulsions, salivation, diarrhea, or lethargy. Somatic motor activity and general behavior patterns were recorded daily to identify potential toxic effects. Individual body weights were measured at baseline (day 0) and at weekly intervals (days 7 and 14) to determine mean body weight gain throughout the study period.

#### 4.7.4. Locomotor Activity Test

Animals were placed in a black polyvinyl Y-maze (arms: 40 cm L × 12 cm H; width: 10 cm; top: 3 cm; bottom) to evaluate locomotor performance. After an initial 8 min acclimation phase, activity was recorded for one hour post-treatment. Locomotion was quantified by counting the number of entries into the maze arms from the central starting point.

#### 4.7.5. Modified Y-Maze Test

Spatial memory was evaluated using a modified Y-maze equipped with a removable black partition to occlude specific arms. During the sample phase, mice were restricted to two arms and allowed to explore for 5 min. To assess short-term memory, a test phase was conducted 30 min later with all three arms accessible for 5 min. Long-term memory was subsequently evaluated 24 h later using the same open-arm configuration. To eliminate olfactory cues, the maze and partition were thoroughly sanitized with 70% ethanol between trials. An entry was recorded when a mouse progressed at least 10 cm into an arm. The preference for the novel (unfamiliar) arm was quantified using the following equation:

(1) $$Unfamiliar\;arm\;exploration\;\left(\%\right)=\frac{Number\;of\;unfamiliar\;arm\;entry}{Number\;of\;all\;arms\;entry}\times 100$$

#### 4.7.6. Novel Object Recognition Test

The NOR test was conducted in an open-field box (52 × 52 × 40 cm). One day prior to testing, mice underwent a 5 min habituation period to explore the empty apparatus. In the acquisition phase, two identical white cylindrical objects (familiar, F) were positioned in opposite corners, 10 cm from the walls, and mice were permitted to explore them for 5 min. Following this, one familiar object was replaced with a novel white rectangular object (N). During the test phase, mice were reintroduced to the arena for 5 min to assess short-term memory (30 min delay) and long-term memory (24 h delay). To eliminate olfactory cues, the arena and objects were thoroughly sanitized with 70% ethanol between trials. Exploration was defined as the snout directed toward an object within a 3 cm radius and was timed via stopwatch. The discrimination index (DI) was calculated using the following formula:

(2) $$Discrimination\;index\;\left(DI\right)=\frac{\left(TN-TF\right)}{\left(TN+TF\right)}\times 100$$

TN refers to time spent with a new object, whereas TF refers to time spent with a familiar object.

#### 4.7.7. Morris Water Maze Test

Spatial learning and memory were assessed using the Morris water maze (MWM), consisting of a black circular pool filled with water (°C) to a depth of 30 cm. A black escape platform was submerged below the water surface in the center of a designated target quadrant. During the acquisition phase, mice underwent five consecutive days of training. In each session, mice were released from various quadrants and allowed 60 s to locate the hidden platform; those failing to do so were guided to it and permitted to remain there for 10 s. On day 6, a probe test was conducted by removing the platform. Mice were allowed to swim freely for 60 s, and the time spent in the target quadrant was recorded as a measure of spatial memory retention.

### 4.8. Identification of Chemical Constituents

Phytochemical profiling of TSB was conducted by UHPLC coupled with high-resolution Orbitrap mass spectrometry (Thermo Fisher Scientific Inc., Bangkok, Thailand). Chromatographic separation employed a Hypersil BDS C18 column (50 × 2.1 mm, 2.4 µm) at 25 °C using a binary mobile phase of 0.1% aqueous formic acid (A) and methanol (B) at 0.5 mL/min. The elution program began isocratically at 50% B for 2 min, followed by a linear gradient to 100% B over 8 min, held for 4 min, then re-equilibrated at 50% A for 3 min. UV detection was recorded at 254 and 337 nm (spectral range 200–800 nm), and the injection volume was 2 µL.

Mass spectrometric data were acquired in both positive and negative ionization modes via heated electrospray ionization (HESI), with spray voltages of 3500 V and 2500 V, respectively. Nitrogen served as the auxiliary gas (sheath: 60 Arb, auxiliary: 15 Arb, sweep: 2 Arb), with both the ion transfer tube and vaporizer set at 350 °C. Full scan spectra were collected over 100–1000 *m*/*z* at 60,000 resolution (RF Lens 70%), with data-dependent MS^2^ triggered at 5.0 × 10^5^ intensity threshold (isolation window 1.5 *m*/*z*, Orbitrap resolution 15,000). Internal calibration was performed using EASY-IC™. Data were processed with Chromeleon™ and FreeStyle software version 1.8.65.0, and compound identification was achieved by comparison with the NIST and mzCloud databases and published literature.

### 4.9. UHPLC-UV Quantification of Piperine

For the quantification of piperine in TSB extract, the same chromatographic system as described in LC-MS/MS was used. The UV at 337 nm, which is the maximum absorbance of piperine, was recorded. Piperine standard was accurately weighed and dissolved in methanol to achieve a concentration of 1 mg/mL. Working standard solutions in the concentration of 3.9–500 µg/mL were prepared by diluting the stock standard solution. The TSB sample was prepared at a concentration of 2 mg/mL in methanol. Prior to the injection, the sample was filtered with a 0.22 µm nylon membrane filter. The analysis was done in triplicate.

### 4.10. Statistical Analysis

All data are expressed as mean ± standard deviation (SD) for in vitro experiments and mean ± standard error of the mean (SEM) for in vivo studies. Statistically significant differences among group means were determined using one-way analysis of variance (ANOVA) followed by Tukey’s post hoc test for multiple comparisons. All statistical analyses were conducted using IBM SPSS Statistics software (version 27.0), with significance thresholds set at * *p* < 0.05 and ** *p* < 0.01.

## 5. Conclusions

This study provides the first systematic scientific evaluation of TSB, a classical Thai polyherbal formulation, against AD. TSB demonstrated significant antioxidant, acetylcholinesterase inhibitory, and anti-amyloid-β aggregation activities, along with neuroprotective effects against oxidative stress-induced neuronal damage in SH-SY5Y cells. Acute oral toxicity assessment confirmed a favorable safety profile with an LD_50_ exceeding 2000 mg/kg. In vivo behavioral studies further validated the cognitive-enhancing potential of TSB, demonstrating significant reversal of scopolamine-induced memory deficits across spatial and recognition memory domains. LC-MS/MS analysis identified piperine as the dominant constituent and primary bioactive driver, with its high and reproducible content supporting its use as a chemical marker for future quality standardization of the formulation. Taken together, these findings suggest that TSB exerts multi-targeted effects against AD pathology through complementary mechanisms, supporting its potential as a multi-component traditional medicine-based therapeutic candidate. Future studies incorporating transgenic AD models, in vivo target validation, and pharmacokinetic profiling of key alkaloids will be essential to further substantiate the translational relevance and clinical potential of TSB.

## Figures and Tables

**Table 1:** The total phenolic and flavonoid contents of Tri-Sannibat-Phol (TSB) extract and its plant components. Data are presented as the mean ± SD from three independent experiments (*n* = 3).

Extracts	Total Phenolic Content (mg Gallic Acid Equivalent/g Extract)	Total Flavonoid Content (mg Quercetin Equivalent/g Extract)
TSB	59.79 ± 8.05	73.01 ± 12.45
*P. retrofractum*	38.56 ± 10.24	50.89 ± 15.63
*O. tenuiflorum*	110.54 ± 11.43	19.04 ± 3.03
*P. nigrum*	104.89 ± 13.76	57.34 ± 13.84

**Table 2:** In vitro antioxidant, acetylcholinesterase (AChE) inhibitory, and amyloid-β (Aβ) aggregation inhibitory activities of the TSB extract and its constituent medicinal plants. Data are presented as the mean ± SD from three independent experiments (*n* = 3). Trolox, donepezil, and curcumin were used as the positive controls for the antioxidant, AChE inhibitory, and Aβ aggregation inhibition assays, respectively.

Extracts and Standard	ABTS	AChE Inhibition	Aβ Aggregation
TSB	237.3 ± 6.33	242.96 ± 0.87	69.25 ± 6.07
*P. retrofractum*	446.41 ± 9.04	333.52 ± 3.52	>200
*O. tenuiflorum*	79.57 ± 8.32	260.76 ± 4.07	57.30 ± 5.88
*P. nigrum*	63.82 ± 8.63	251.51 ± 3.74	53.00 ± 0.50
Trolox (µM)	43.67 ± 0.77	ND	ND
Donepezil	-	0.07 ± 0.00	-
Curcumin	ND	ND	10.75 ± 1.09

Note: ND (not determined) indicates that the corresponding assay was not performed because it was not applicable to the reference compound.

**Table 3:** Mortality and clinical signs observations of animals treated with TSB extract in sighting and main study.

Groups	Study Type	N (Live)	N (Dead)	Number of Mice Died Within 14th Day	Clinical Signs	Percentage of Mortality
Control	Sightseeing study	1	-	0	Normal	0
	Main study	2	-			
		3	-			
		4	-			
		5	-			
TSB 5 mg/kg	Sightseeing study	1	-	0	Normal	0
	Main study	2	-			
		3	-			
		4	-			
		5	-			
TSB 50 mg/kg	Sightseeing study	1	-	0	Normal	0
	Main study	2	-			
		3	-			
		4	-			
		5	-			
TSB 300 mg/kg	Sightseeing study	1	-	0	Normal	0
	Main study	2	-			
		3	-			
		4	-			
		5	-			
TSB 2000 mg/kg	Sightseeing study	1	-	0	Normal	0
	Main study	2	-			
		3	-			
		4	-			
		5	-			

## Data Availability

The original contributions presented in this study are included in the article/Supplementary Materials. Further inquiries can be directed to the corresponding author.

## Supplementary Materials

The following supporting information can be downloaded at: https://www.mdpi.com/article/10.3390/ph19071063/s1. Table S1: Peak area normalization of identified constituents relative to piperine in the UHPLC chromatogram of TSB extract (2 mg/mL) at 254 nm.; Table S2: Identification of major components of TSB extract by LC-MS/MS; Figure S1: Quantitative analysis of piperine in TSB extract (2 mg/mL) by using UHPLC-UV; Figures S2–S20: Mass spectra of all identified compounds.

## Abbreviations

The following abbreviations are used in this manuscript:

ABTS	2,2′-azino-bis(3-ethylbenzothiazoline-6-sulfonic acid)
AChE	Acetylcholinesterase
AD	Alzheimer’s disease
Aβ	Amyloid beta
DNP	Donepezil
H_2_O_2_	Hydrogen peroxide
MWM	Moris water maze
NOR	Novel object recognition
SCOP	Scopolamine
TPC	Total phenolic content
TFC	Total flavonoid content
TTM	Traditional Thai Medicine
TSB	Tri-Sannibat-Phol
