# Supplementary material for: Multi-Targeted Anti-Alzheimer’s Effects of Tri-Sannibat-Phol: Biological Evaluation, Behavioral Validation, and LC-MS/MS Phytochemical Profiling
Source: Pharmaceuticals (Basel). 2026 Jul 9;19(7):1063. doi: 10.3390/ph19071063 (PMC13415300; doi:10.3390/ph19071063)
Supplement: Supplementary file 1 [file pharmaceuticals-19-01063-s001.zip › pharmaceuticals-4359944-supplementary.pdf]

Supplementary data

Multi-Targeted Anti-Alzheimer's Effects of Tri-Sannibat-Phol: Biological Evaluation, Behavioral Validation, and LC-MS/MS Phytochemical Profiling

Pitchayakarn Takomthong[1], Pornthip Waiwut[2], Sumet Kongkiatpaiboon[3], Khemjira Phemphunananchai[1], Chantana Boonyarat[1,4,*]

[1] *Faculty of Pharmaceutical Sciences, Khon Kaen University, Khon Kaen 40002, Thailand; ppitcha.t@gmail.com (P.T.), khemjira_ph@kkumail.com (K.P.), chaboo@kku.ac.th (C.B.).*

[2] *Faculty of Pharmaceutical Sciences, Ubon Ratchathani University, Ubon Ratchathani 34190, Thailand; pwaiwut79@yahoo.com (P.W.).*

[3] *Drug Discovery and Development Center, Office of Advanced Science and Technology, Thammasat University (Rangsit campus), Pathum Thani 12121, Thailand;* sumet_k@tu.ac.th *(S.K.).*

[4] *Center for Research and Development of Herbal Health Products, Khon Kaen University, Khon Kaen 40002, Thailand.*

* *Correspondence: chaboo@kku.ac.th; Tel.: +66-81-3073313, +66-43-202305*

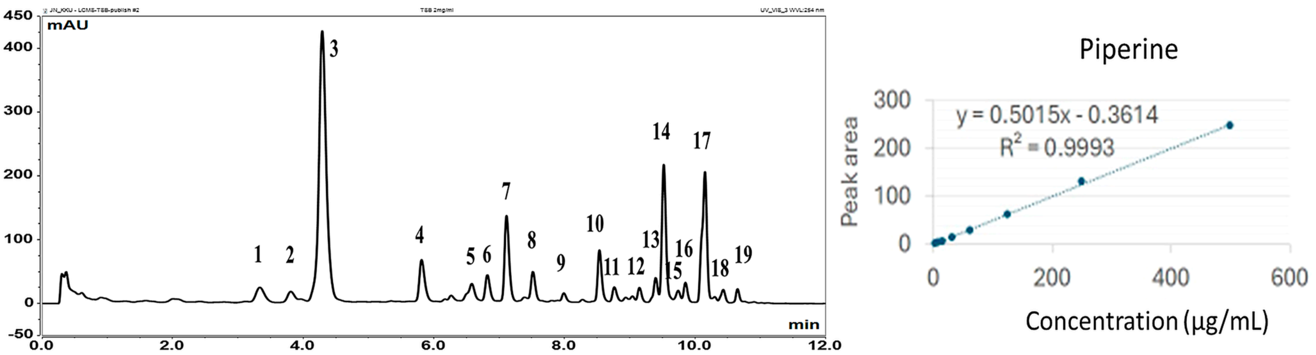

Figure S1 Quantitative analysis of piperine in TSB extract (2 mg/mL) by using UHPLC-UV.

Table S1. Peak area normalization of identified constituents relative to piperine in the UHPLC chromatogram of TSB extract (2 mg/mL) at 254 nm.

Peak No.	Identification	Peak area at 254 nm	Peak area ratio at 254 nm (compared to piperine)
1	Dihydropiperyline<br>$C_{16}H_{19}O_3N$<br>MW = 273	3.9455	0.0692
2	Piperanine<br>$C_{17}H_{21}O_3$<br>MW = 287<br>$C_{17}H_{21}O_3N$	2.7967	0.0491
3	Piperine<br>$C_{17}H_{19}O_3$<br>$C_{17}H_{19}O_3N$<br>MW = 285	57.0080<br>(17.61 % w/w in TSB extract)	1.0000
4	Pellitorine<br>$C_{14}H_{25}ON$<br>MW = 223	6.1652	0.1081
5+6	unknown<br>$C_{20}H_{27}O_3N$<br>MW = 329	3.1476	0.0552
	unknown<br>$C_{21}H_{25}O_3N$<br>MW = 339		
7	Dehydropipernonaline<br>$C_{21}H_{25}O_3N$<br>MW = 339	3.2866	0.0577
8	Pipernonaline<br>$C_{21}H_{27}O_3N$<br>MW = 341	10.6775	0.1873
9	Pipercide or Retrofractamide B<br>$C_{22}H_{29}O_3N$<br>MW = 355	3.1059	0.0545
10	Piperundecalidine<br>$C_{23}H_{29}O_3$<br>MW = 367<br>$C_{23}H_{29}O_3N$	1.3877	0.0243
11	unknown	5.6460	0.0990

Peak No.	Identification	Peak area at 254 nm	Peak area ratio at 254 nm (compared to piperine)
	$C_{24}H_{33}O_3N$<br>MW = 383		
12	N-isobutyl-2,4-10-hexadecatrienamide<br>$C_{20}H_{35}ON$<br>MW = 305	1.5972	0.0280
13	unknown<br>$C_{21}H_{35}ON$<br>MW=317	1.2962	0.0227
14	unknown<br>$C_{20}H_{37}ON$<br>MW = 307	1.7991	0.0316
15	N-isobutyl-2,4,12-octadecatrienamide or N-isobutyl-2,4,10-octadecatrienamide<br>$C_{22}H_{39}ON$<br>MW = 333	16.7994	0.2947
16	1-(piperidmyl)-2,4,12-octadecatrien-1-one<br>$C_{23}H_{39}ON$<br>MW = 345	2.1328	0.0374
17	N-isobutyl-2,4,14-eicosatrienamide<br>$C_{24}H_{43}ON$<br>MW = 361	17.0126	0.2984
18	1-(piperidinyl)-2,4,14-eicosatrien-1-one<br>$C_{25}H_{43}ON$<br>MW = 373	1.3371	0.0235
19	N-isobutyl-2,4-eicosadienamide<br>$C_{24}H_{45}ON$<br>MW = 363	1.4427	0.0253

Table S2. Identification of major components of TSB extract by LC-MS/MS

Peak No.	Retention time (min)	MS mode	MS1	MS2	Results
1	3.337	Positive	274.1436<br>$C_{16}H_{20}O_3N$<br>Calc for $[M+H]^+$ = 274.14377	274.1436 -> 201.0546, 135.0440, 115.0542	4,5-dihydropiperyline<br>$C_{16}H_{19}O_3N$<br>MW = 273
2	3.619	Positive	288.1591<br>$C_{17}H_{22}O_3N$<br>Calc for $[M+H]^+$ = 288.15942	288.1591 -> 202.0776, 135.0440, 138.0913	Piperanine<br>$C_{17}H_{21}O_3N$<br>MW = 287
			310.1411<br>$C_{17}H_{21}O_3NNa$<br>Calc for $[M+Na]^+$ = 310.14136	310.1411 -> 202.0777	
3	4.293	Positive	286.1434<br>$C_{17}H_{20}O_3N$<br>Calc for $[M+H]^+$ = 286.14377	286.1434 -> 201.0545, 135.0440, 115.0541	Piperine<br>$C_{17}H_{19}O_3N$<br>MW=285
4	5.813	Positive	224.2007<br>$C_{14}H_{26}ON$<br>Calc for $[M+H]^+$ = 224.20089	224.2007 -> 202.0778, 168.1384, 151.1117	Pellitorine<br>$C_{14}H_{25}ON$<br>MW = 223
			246.1826<br>$C_{14}H_{25}ONNa$<br>Calc for $[M+Na]^+$ = 246.18284	246.1826 -> 202.0776	
5+6	6.580<br><br>2 overlapped compounds	Positive	330.2063<br>$C_{20}H_{28}O_3N$<br>Calc for $[M+H]^+$ = 330.20637	330.2063 -> 229.1223, 202.0778, 161.0599, 135.0441	unknown<br>$C_{20}H_{27}O_3N$<br>MW = 329
			340.1906<br>$C_{21}H_{26}O_3N$<br>Calc for $[M+H]^+$ = 340.19072	340.1905 -> 255.1017, 227.1066, 202.0778, 112.0756	unknown<br>$C_{21}H_{25}O_3N$<br>MW = 339
7	6.820	Positive	340.1904<br>$C_{21}H_{26}O_3N$<br>Calc for $[M+H]^+$ = 340.19072	340.1905 -> 202.0779, 179.1305, 161.0597, 131.0491, 112.0756	Dehydropipernonaline<br>$C_{21}H_{25}O_3N$<br>MW = 339

Peak No.	Retention time (min)	MS mode	MS1	MS2	Results
			362.1725<br>$C_{21}H_{25}O_3NNa$<br>Calc for $[M+Na]^+$ = 362.17266	362.1725 -> 202.0779	
8	7.113	Positive	342.2062<br>$C_{21}H_{28}O_3N$<br>Calc for $[M+H]^+$ = 342.20637	342.2060 -> 229.1224, 135.0441, 161.0597	Pipernonaline<br>$C_{21}H_{27}O_3N$<br>MW = 341
			364.1882<br>$C_{21}H_{27}O_3NNa$<br>Calc for $[M+Na]^+$ = 364.18831	364.1882 -> 202.0778	
9	7.517	Positive	356.2219<br>$C_{22}H_{30}O_3N$<br>Calc for $[M+H]^+$ = 356.22202	356.2219 -> 255.1378, 202.0777, 161.0597, 135.0440	Pipercide or Retrofractamide B<br>$C_{22}H_{29}O_3N$<br>MW = 355
			378.2039<br>$C_{22}H_{29}O_3NNa$<br>Calc for $[M+Na]^+$ = 378.20396	378.2038 -> 202.0777, 238.5946	
10	7.990	Positive	368.2218<br>$C_{23}H_{30}O_3N$<br>Calc for $[M+H]^+$ = 368.22202	368.2219 -> 202.0777, 255.1380, 135.0440	Piperundecalidine<br>$C_{23}H_{29}O_3N$<br>MW = 367
			390.2038<br>$C_{23}H_{29}O_3NNa$<br>Calc for $[M+Na]^+$ = 390.20396	390.2039 -> 202.0775	
11	8.537	Positive	384.2531<br>$C_{24}H_{34}O_3N$<br>Calc for $[M+H]^+$ = 384.25332	384.2531 -> 283.1693, 202.0777, 135.0440	unknown<br>$C_{24}H_{33}O_3N$<br>MW = 383
			406.2351<br>$C_{24}H_{33}O_3NNa$<br>Calc for $[M+Na]^+$ = 406.23527	406.2351 -> 202.0777	
12	8.763	Positive	306.2789<br>$C_{20}H_{36}ON$	N/A	N-isobutyl-2,4,10-hexadecatrienamide

Peak No.	Retention time (min)	MS mode	MS1	MS2	Results
			Calc for $[M+H]^+$ = 306.27914		$C_{20}H_{35}ON$<br>MW = 305
			328.2610<br>$C_{20}H_{35}ONNa$<br>Calc for $[M+Na]^+$ = 328.26109	328.2607 -> 202.0778	
13	9.150	Positive	318.2788<br>$C_{21}H_{36}ON$<br>Calc for $[M+H]^+$ = 318.27914	318.2789 -> 202.0778, 112.0757	unknown<br>$C_{21}H_{35}ON$<br>MW=317
			340.2609<br>$C_{21}H_{35}ONNa$<br>Calc for $[M+Na]^+$ = 340.26109	340.2610 -> 202.0776	
14	9.397	Positive	308.2946<br>$C_{20}H_{38}ON$<br>Calc for $[M+H]^+$ = 308.29479	308.2946 -> 202.0776	unknown<br>$C_{20}H_{37}ON$<br>MW = 307
			330.2766<br>$C_{20}H_{37}ONNa$<br>Calc for $[M+Na]^+$ = 330.27674	330.2764 -> 202.0777	
15	9.520	Positive	334.3120<br>$C_{22}H_{40}ON$<br>Calc for $[M+H]^+$ = 334.31044	334.3103 -> 202.0780	N-Isobutyl-2,4,12-octadecatrienamide<br>$C_{22}H_{39}NO$<br>MW=333
			356.2923<br>$C_{22}H_{39}ONNa$<br>Calc for $[M+Na]^+$ = 356.29239	356.2922 -> 202.0777	
			667.6133<br>$C_{44}H_{79}O_2N_2$<br>Calc for $[M+H]^+$ = 667.61361	667.6133 -> 334.3104, 202.0777	Dimer
			689.5952<br>$C_{44}H_{78}O_2N_2Na$<br>Calc for $[M+Na]^+$ = 689.59555	689.5952 -> 356.2923, 202.0777	

Peak No.	Retention time (min)	MS mode	MS1	MS2	Results
16	9.853	Positive	346.3101 $C_{23}H_{40}ON$ Calc for $[M+H]^+$ = 346.31044	346.3102 -> 202.0777	1-(piperidinyl)-2,4,12-octadecatrien-1-one $C_{23}H_{39}ON$ MW = 345
			368.2922 $C_{23}H_{39}ONNa$ Calc for $[M+Na]^+$ = 368.29239	368.2922 -> 202.0777	
17	10.153 A dimer was formed during ionization	Positive	362.3416 $C_{24}H_{44}ON$ Calc for $[M+H]^+$ = 362.34174	362.3415 -> 202.0778	N-isobutyl-2,4,14-eicosatrienamide $C_{24}H_{43}ON$ MW = 361
			384.3236 $C_{24}H_{43}ONNa$ Calc for $[M+Na]^+$ = 384.32369	384.3236 -> 202.0779	
			723.6758 $C_{48}H_{86}O_2N_2$ Calc for $[M+H]^+$ = 723.67621	723.6758 -> 362.3417, 202.0778	Dimer
			745.6576 $C_{48}H_{85}O_2N_2Na$ Calc for $[M+Na]^+$ = 745.65815	745.6580 -> 384.3236, 202.0776	
18	10.433	Positive	374.3415 $C_{25}H_{44}ON$ Calc for $[M+H]^+$ = 374.34174	374.3416 -> 202.0777	1-(piperidinyl)-2,4,14-eicosatrien-1-one $C_{25}H_{43}ON$ MW = 373
			396.3235 $C_{25}H_{43}ONNa$ Calc for $[M+Na]^+$ = 396.32369	396.3237 -> 202.0775	
19	10.650	Positive	364.3572 $C_{24}H_{46}ON$ Calc for $[M+H]^+$ = 364.35739	364.3572 -> 202.0776	N-isobutyl-2,4-eicosadienamide $C_{24}H_{45}ON$ MW = 363
			386.3392 $C_{24}H_{45}ONNa$	386.3395 -> 202.0778	

Peak No.	Retention time (min)	MS mode	MS1	MS2	Results
			Calc for $[M+Na]^+$ = 386.33934		
20	6.92	Negative	551.3042 $C_{26}H_{47}O_{12}$ Calc for $[M-H]^-$ = 551.30620	551.3042-> 521.2583, 95.9593, 79.9574	Unknown $C_{26}H_{48}O_{12}$ MW = 552

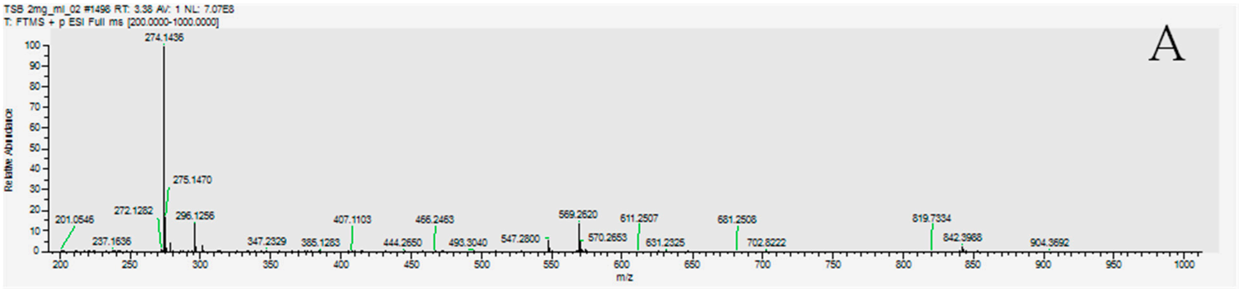

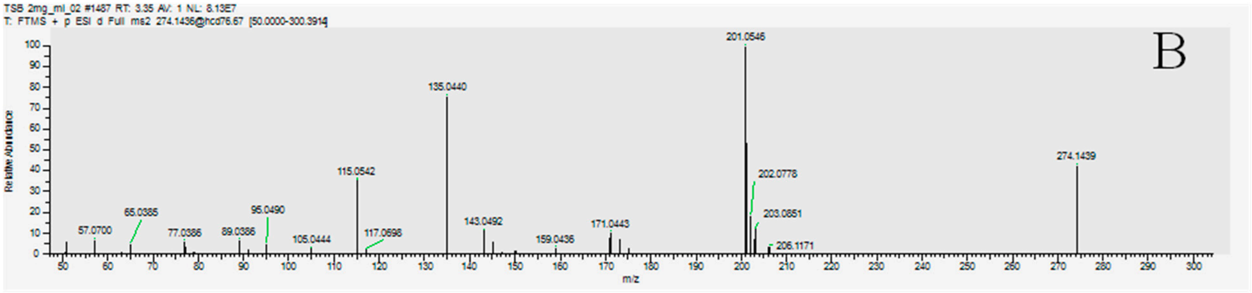

Figure S2 Mass spectra of dihydropiperyline (1) at retention time of 3.337 min. (A) full scan of 200-1000 m/z with positive mode, (B) MS^2 scan in positive mode of precursor of 274.1436 m/z.

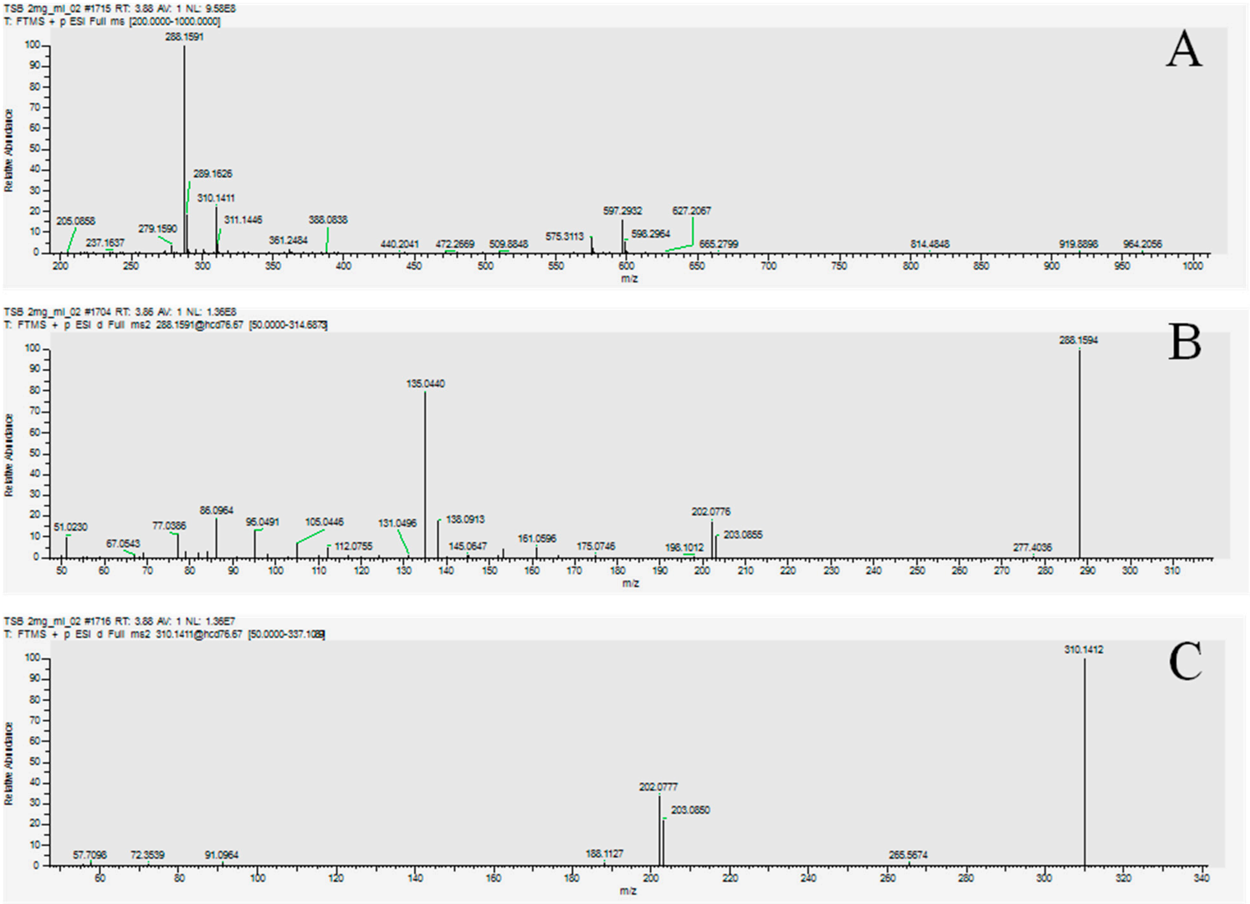

Figure S3 Mass spectra of piperanine (2) at retention time of 3.619 min. (A) full scan of 200-1000 m/z with positive mode, (B) MS^2 scan in positive mode of precursor of 288.1591 m/z, (C) MS^2 scan in positive mode of precursor of 310.1411 m/z.

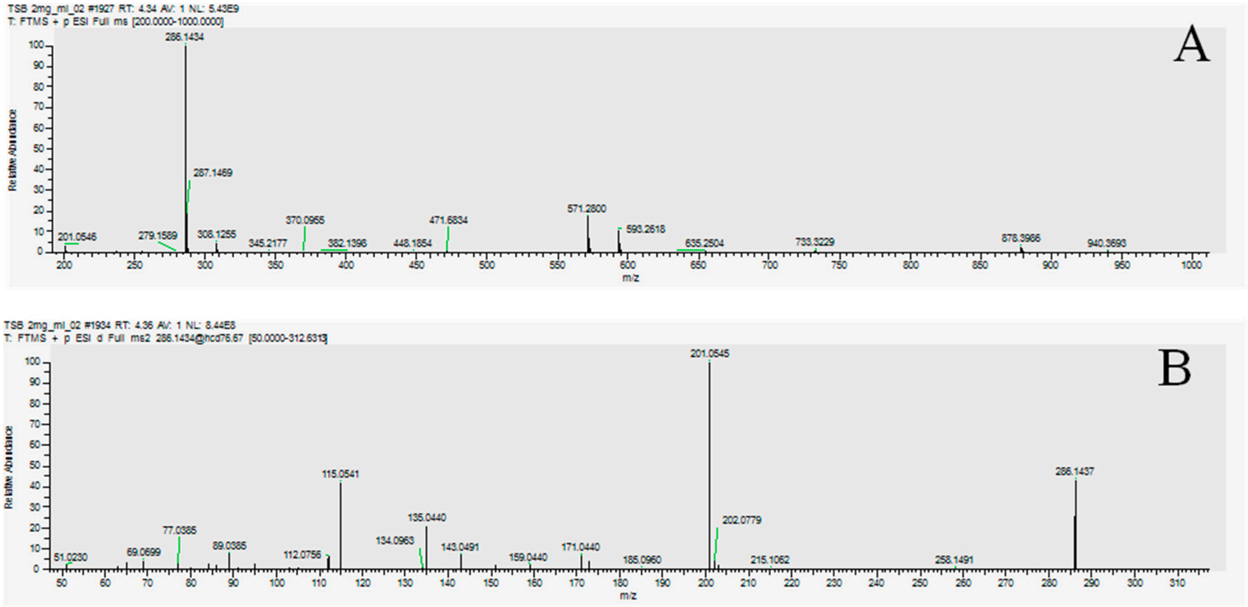

Figure S4 Mass spectra of piperine (3) at retention time of 4.293 min. (A) full scan of 200-1000 m/z with positive mode, (B) MS^2 scan in positive mode of precursor of 286.1434 m/z.

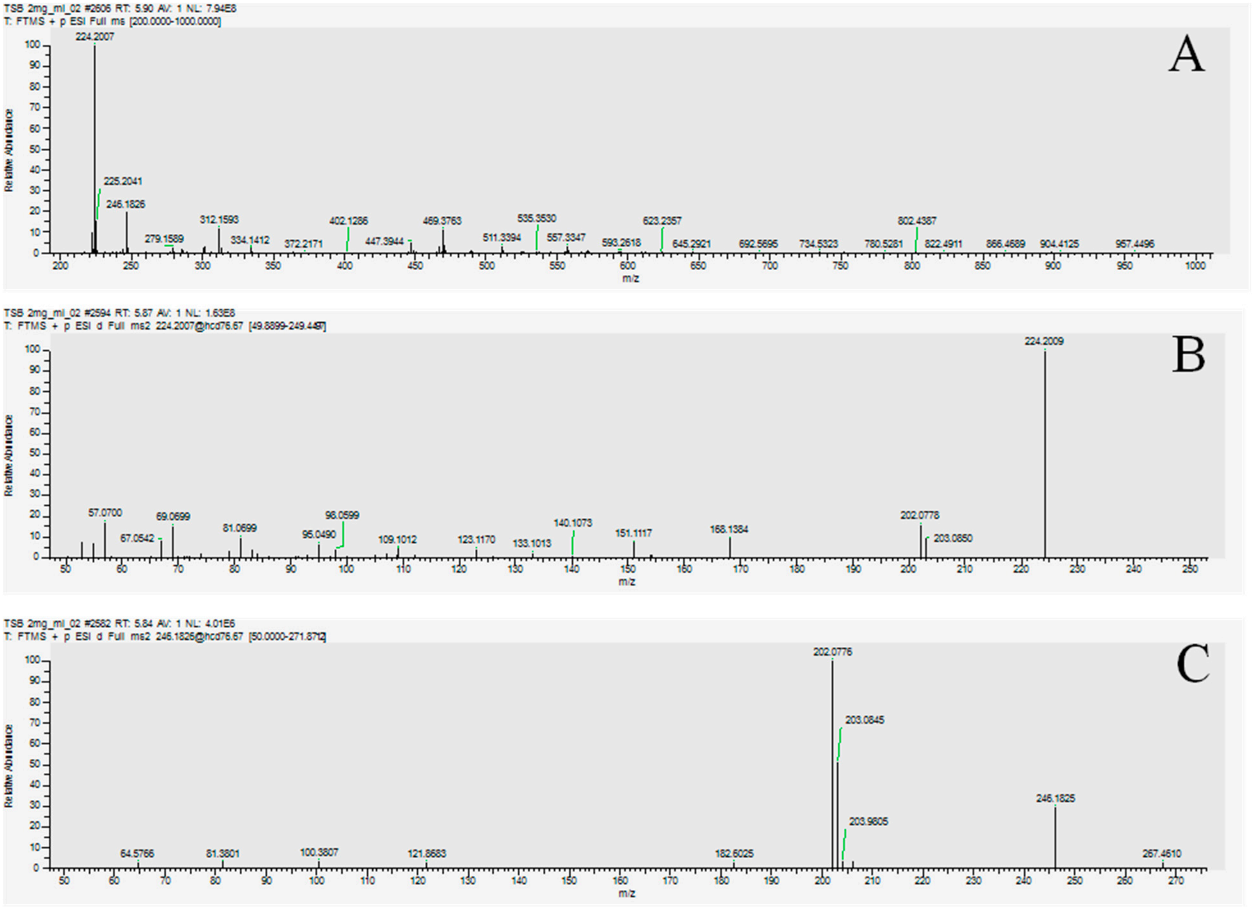

Figure S5 Mass spectra of Pellitorine (4) at retention time of 5.813 min. (A) full scan of 200-1000 m/z with positive mode, (B) MS² scan in positive mode of precursor of 224.2007 m/z, (C) MS² scan in positive mode of precursor of 246.1826 m/z.

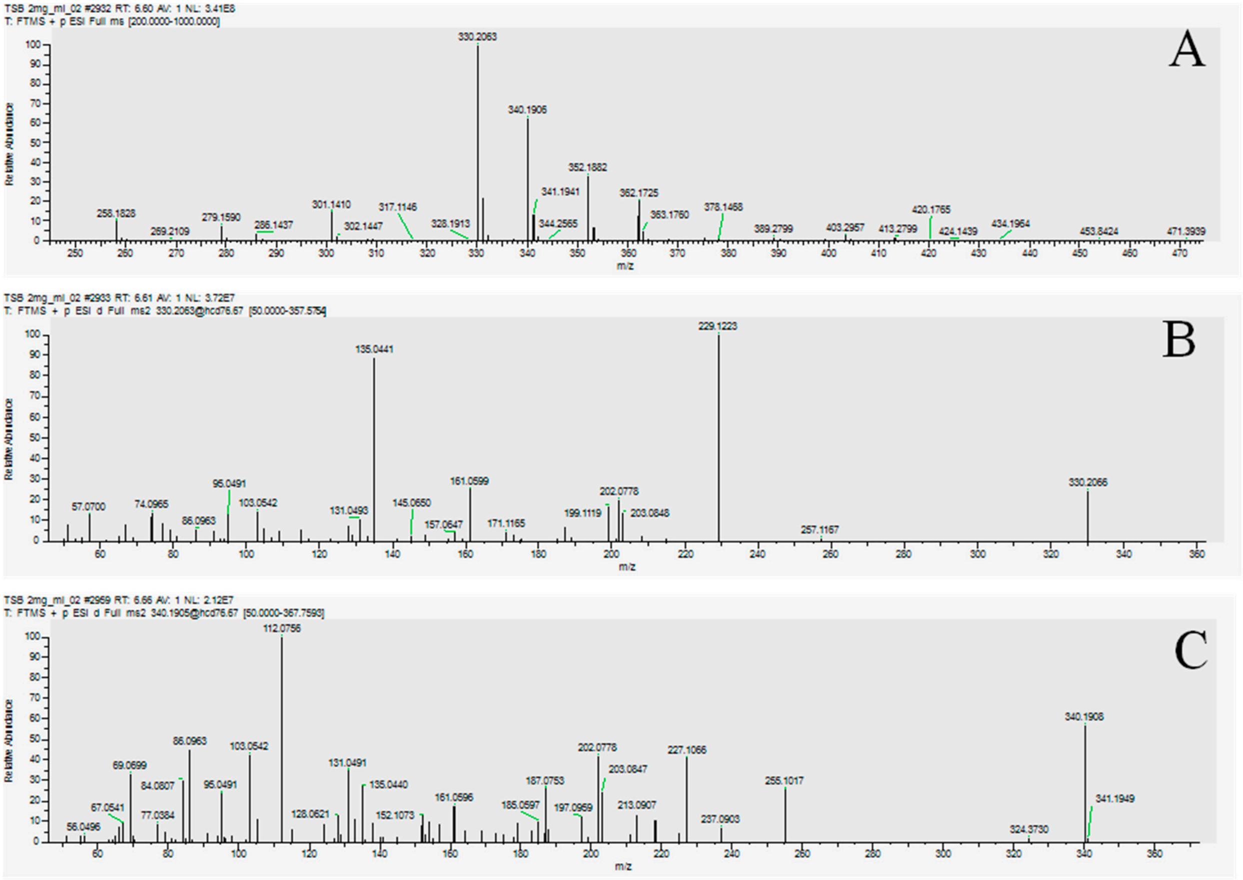

Figure S6 Mass spectra of unknown ($C_{20}H_{27}O_3N$) (5) and unknown ($C_{21}H_{25}O_3N$) (6) at retention time of 6.580 min. (A) full scan of 200-1000 m/z with positive mode, (B) MS^2 scan in positive mode of precursor of 330.2063 m/z, and (C) MS^2 scan in positive mode of precursor of 340.1905 m/z.

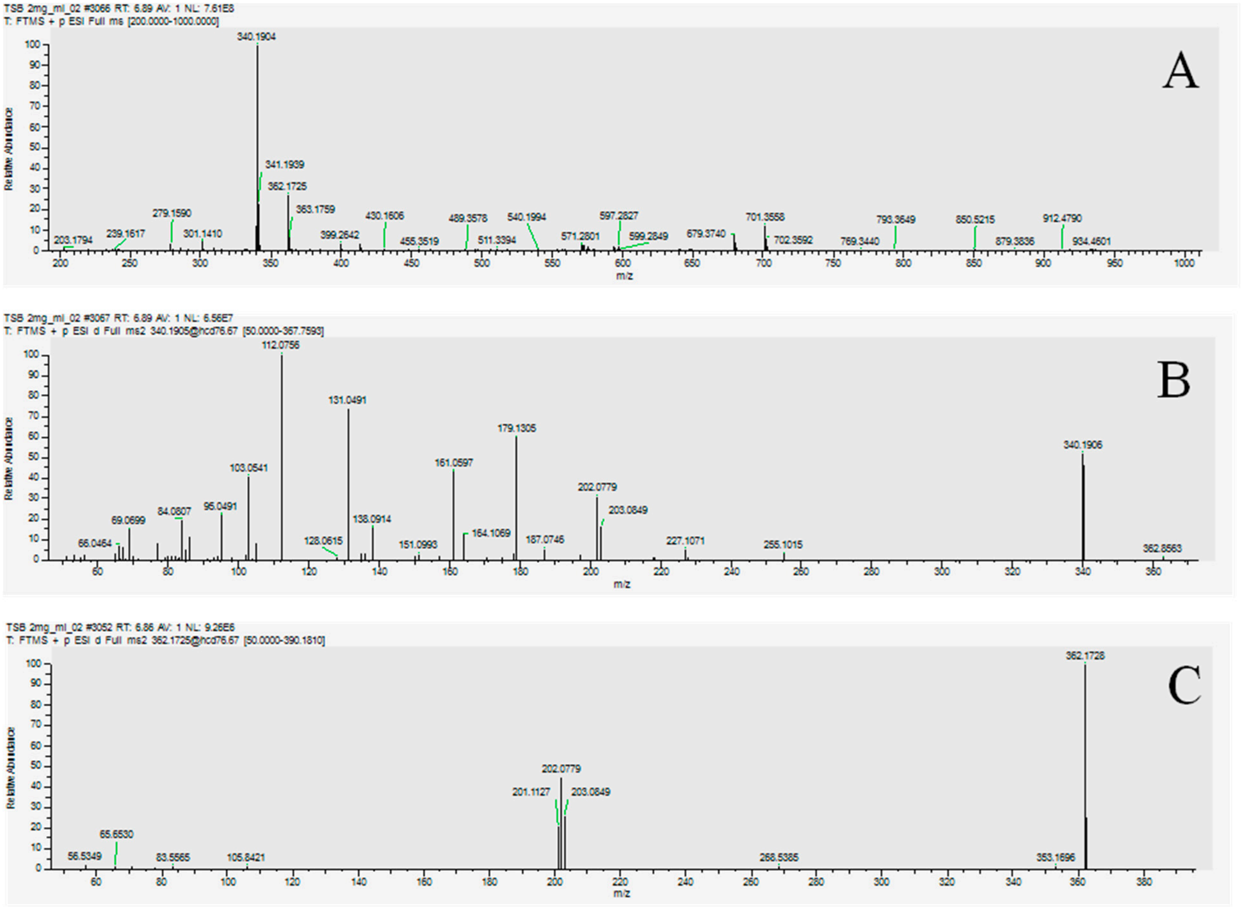

Figure S7 Mass spectra of dehydropipernonaline (7) at retention time of 6.820 min. (A) full scan of 200-1000 m/z with positive mode, (B) MS^2 scan in positive mode of precursor of 340.1904 m/z, (C) MS^2 scan in positive mode of precursor of 362.1725 m/z.

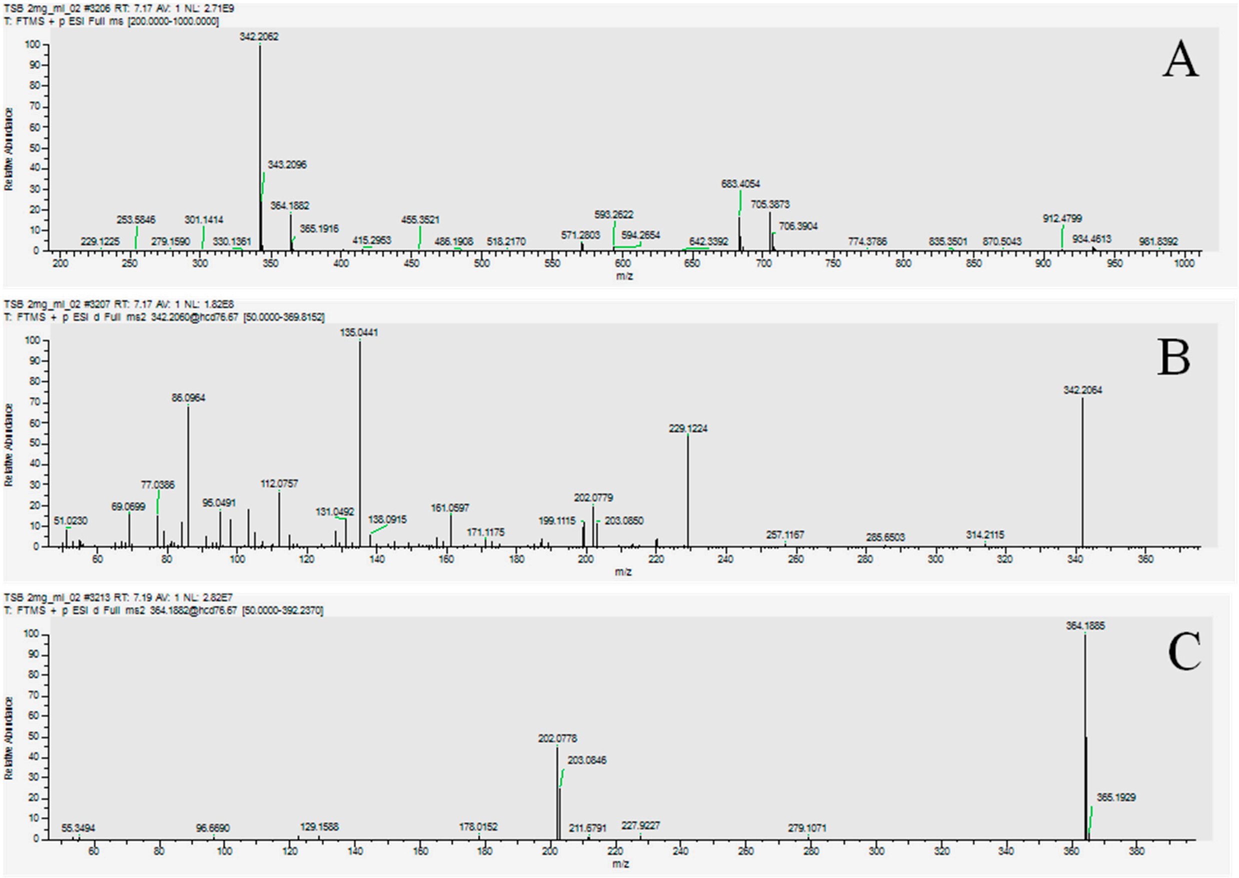

Figure S8 Mass spectra of Pipernonaline (8) at retention time of 7.113 min. (A) full scan of 200-1000 m/z with positive mode, (B) MS^2 scan in positive mode of precursor of 342.2062 m/z, (C) MS^2 scan in positive mode of precursor of 364.1882 m/z.

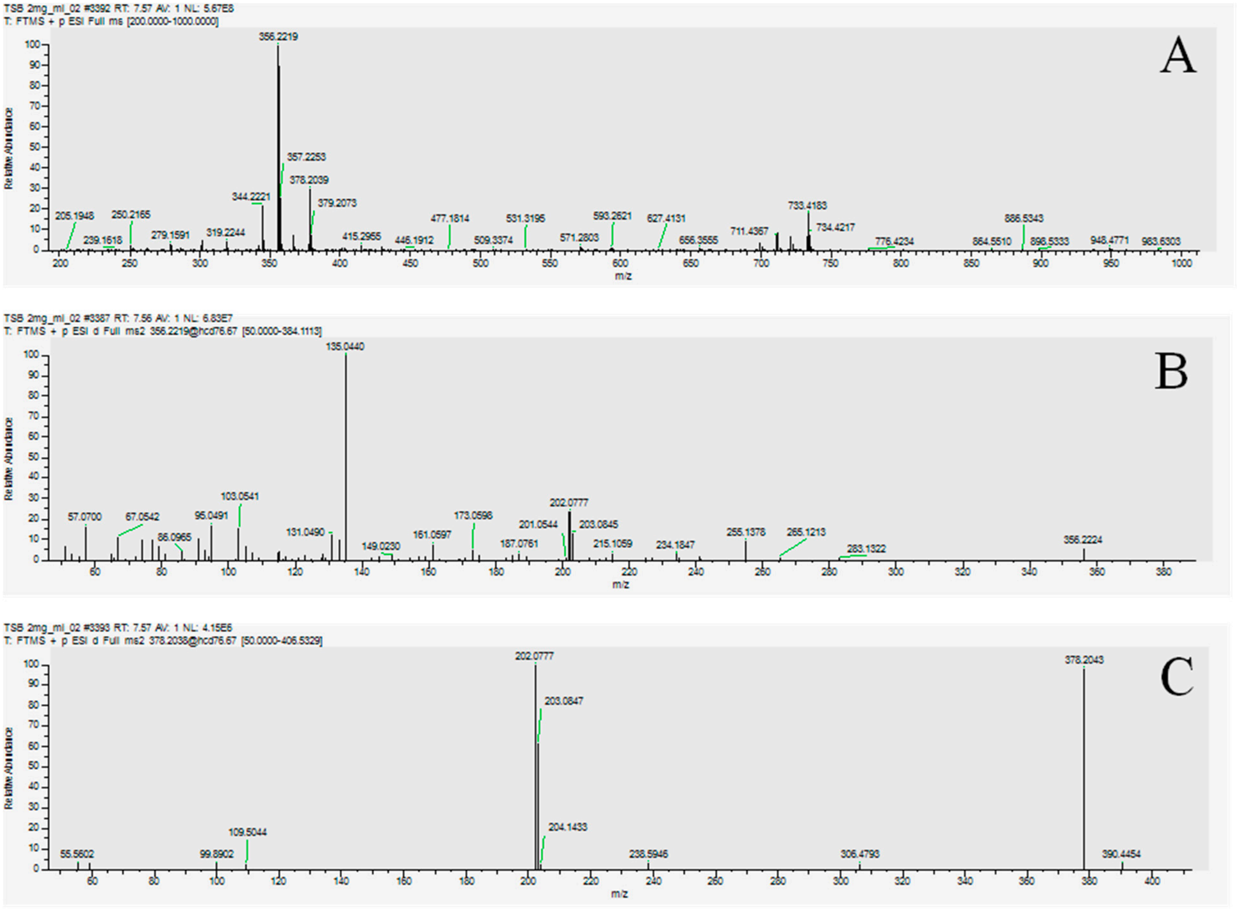

Figure S9 Mass spectra of Pipercide (Retrofractamide B) (9) at retention time of 7.517 min. (A) full scan of 200-1000 m/z with positive mode, (B) MS² scan in positive mode of precursor of 356.2219 m/z, (C) MS² scan in positive mode of precursor of 378.2039 m/z.

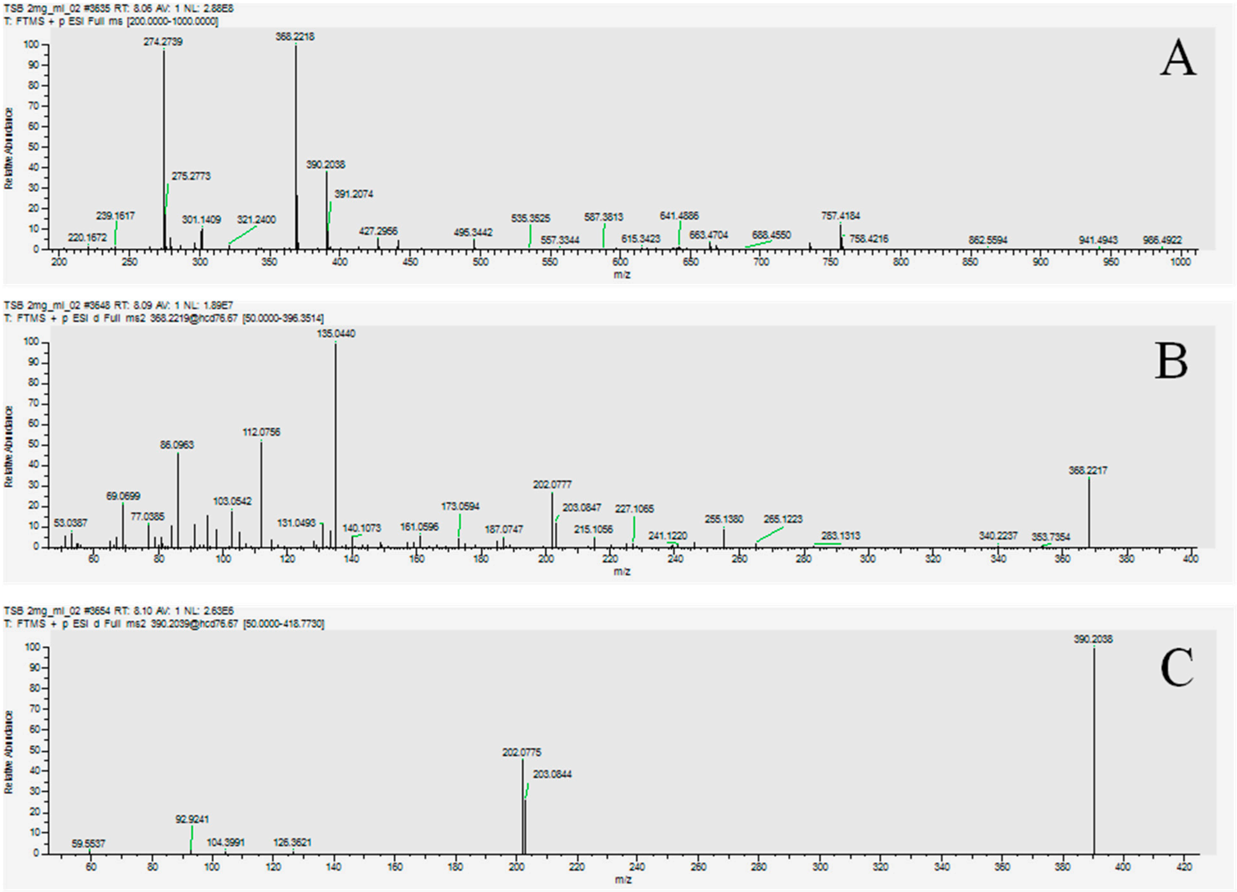

Figure S10 Mass spectra of Piperundecalidine (10) at retention time of 7.990 min. (A) full scan of 200-1000 m/z with positive mode, (B) MS^2 scan in positive mode of precursor of 368.2218 m/z, (C) MS^2 scan in positive mode of precursor of 390.2038 m/z.

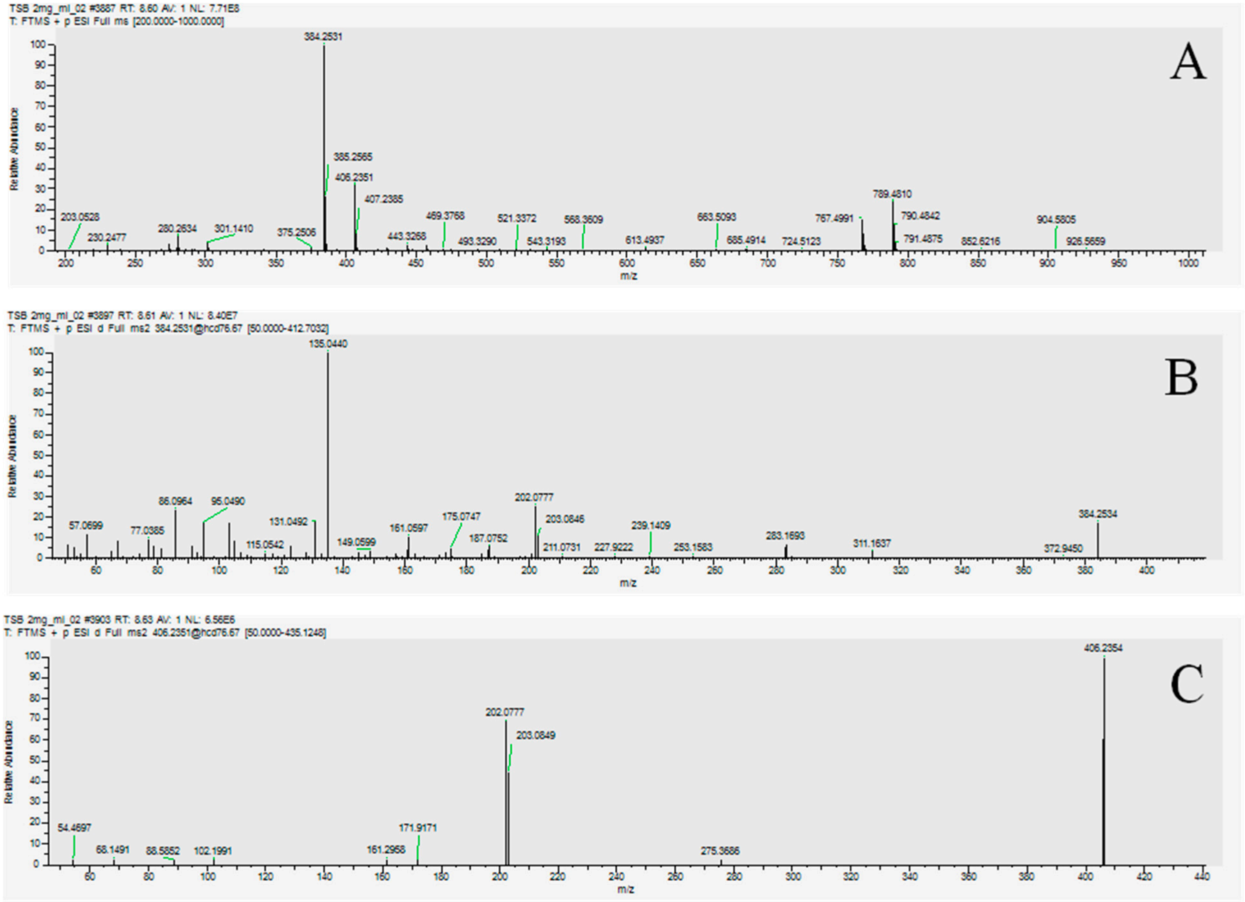

Figure S11 Mass spectra of Unknow ($C_{24}H_{33}O_3N$) (11) at retention time of 8.537 min. (A) full scan of 200-1000 m/z with positive mode, (B) MS^2 scan in positive mode of precursor of 384.2531 m/z, (C) MS^2 scan in positive mode of precursor of 406.2351 m/z.

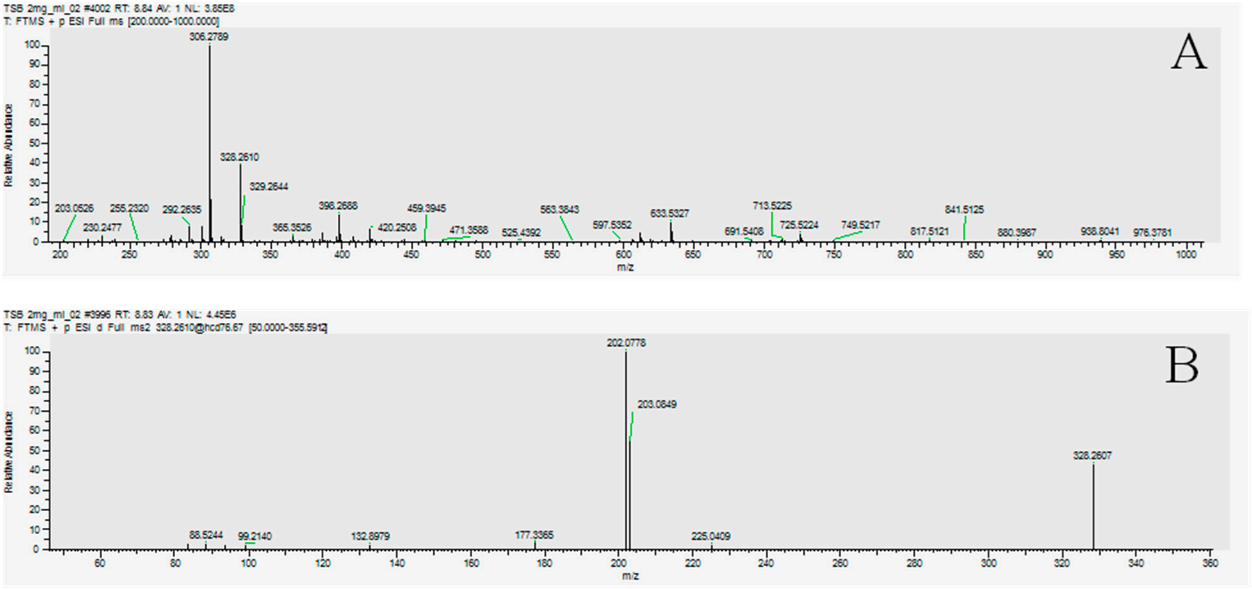

Figure S12 Mass spectra of N-isobutyl-2,4,10-hexadecatrienamide (12) at retention time of 8.763 min. (A) full scan of 200-1000 m/z with positive mode, (B) MS^2 scan in positive mode of precursor of 328.2610 m/z.

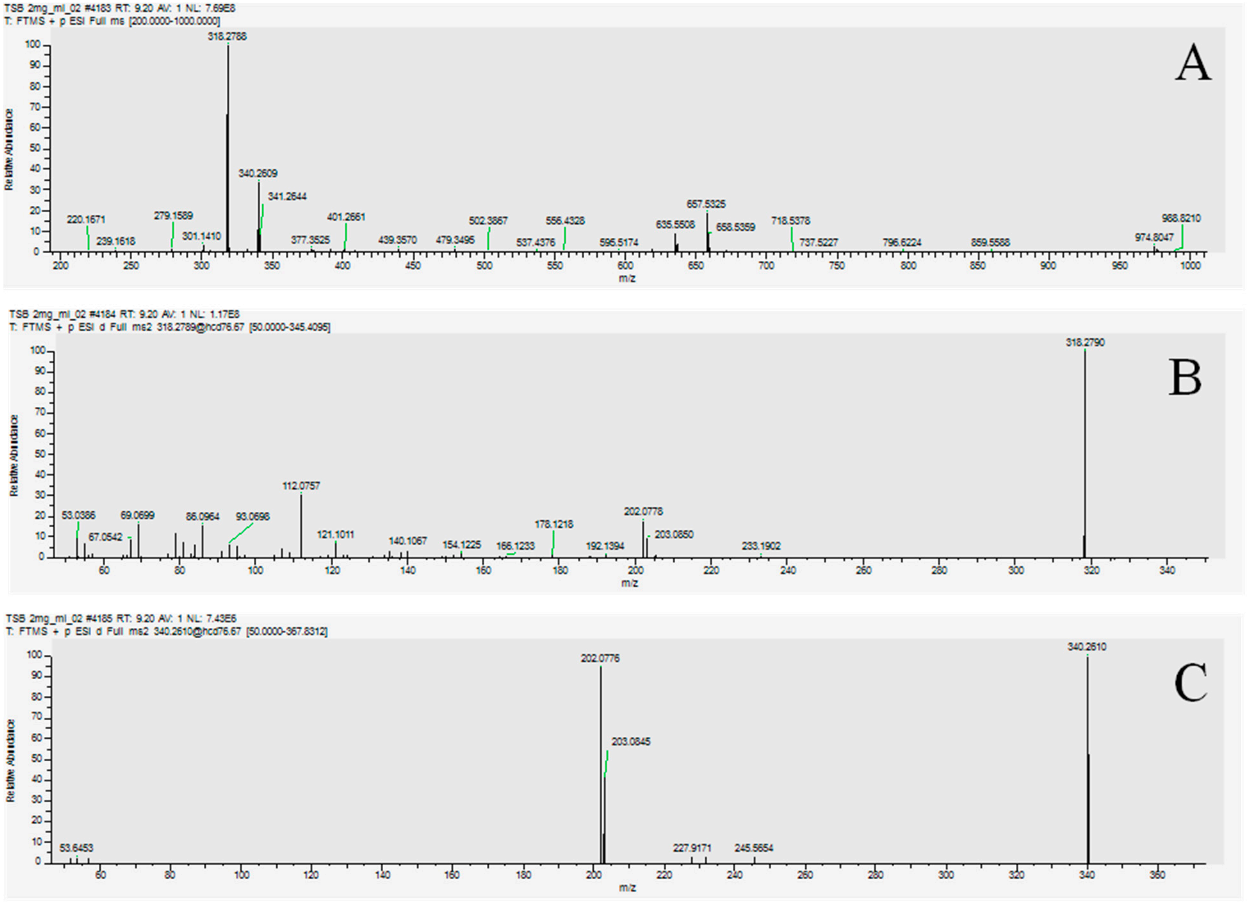

Figure S13 Mass spectra of unknown (C21H35ON) (13) at retention time of 9.150min. (A) full scan of 200-1000 m/z with positive mode, (B) MS[2] scan in positive mode of precursor of 318.2788 m/z, (C) MS[2] scan in positive mode of precursor of 340.2609 m/z.

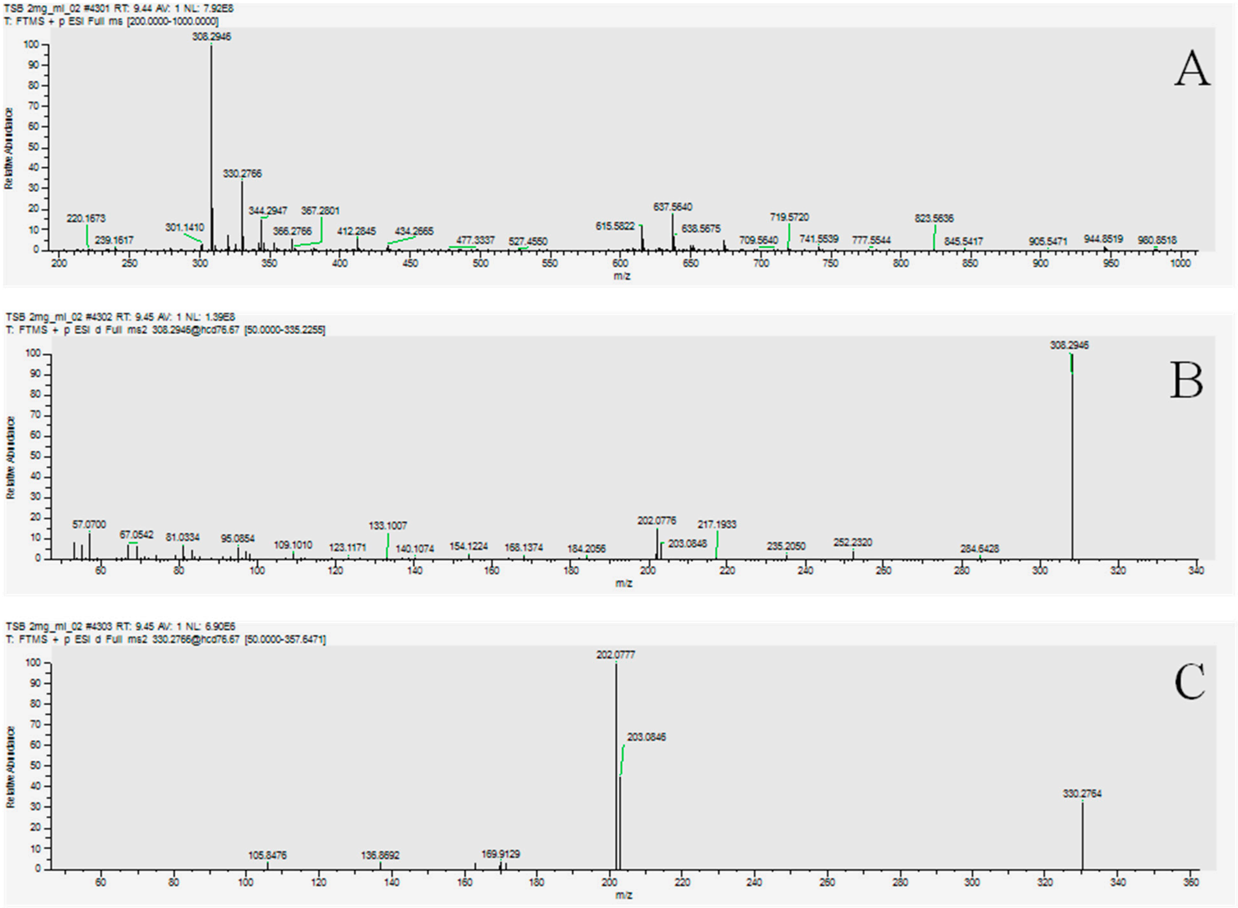

Figure S14 Mass spectra of unknown ($C_{20}H_{37}ON$) (14) at retention time of 9.397 min. (A) full scan of 200-1000 m/z with positive mode, (B) MS^2 scan in positive mode of precursor of 308.2946 m/z, (C) MS^2 scan in positive mode of precursor of 330.2766 m/z.

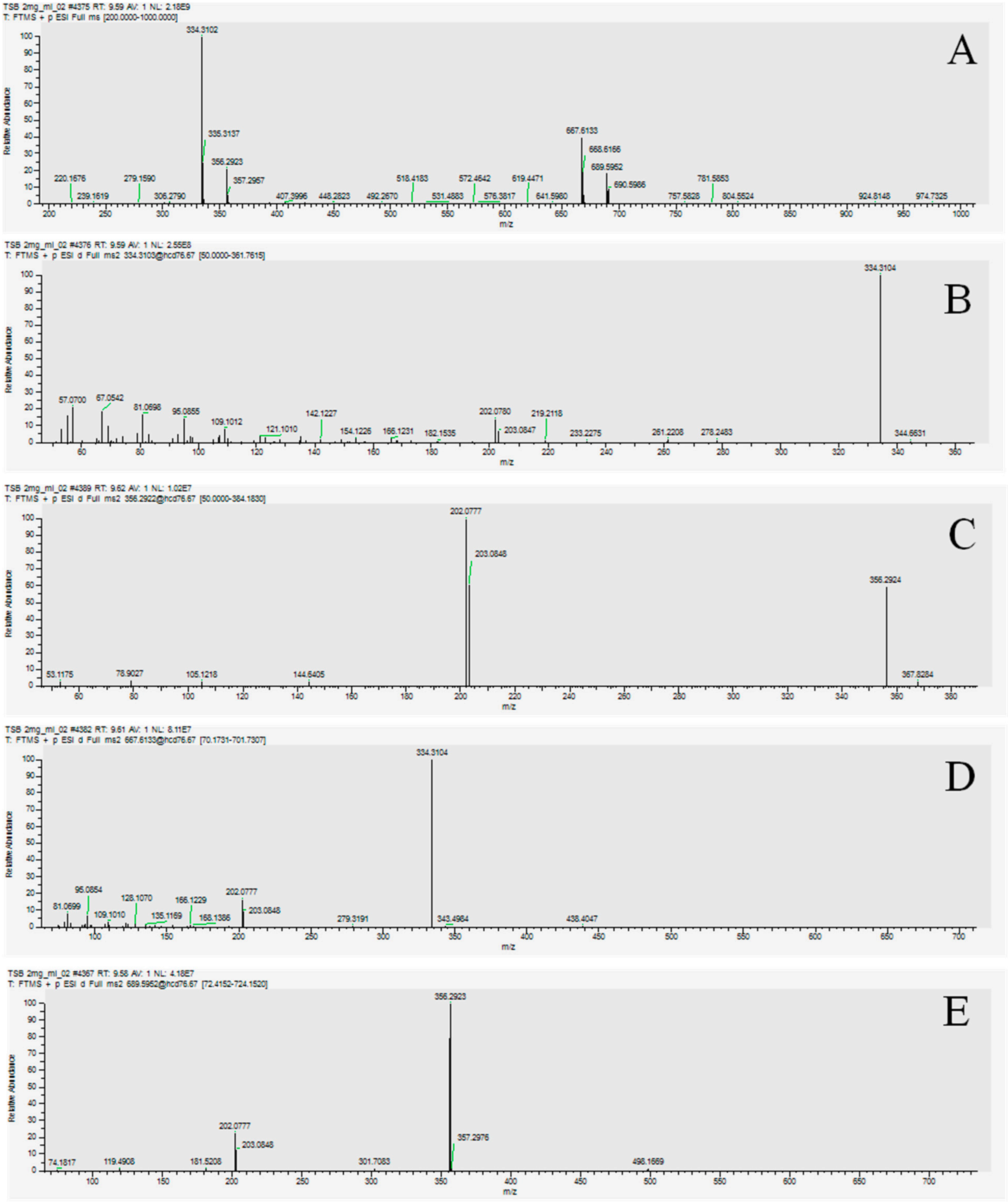

Figure S15 Mass spectra of N-isobutyl-2,4,12-octadecatrienamide (15) at retention time of 9.520 min. (A) full scan of 200-1000 m/z with positive mode, (B) MS^2 scan in positive mode of precursor of 334.3102 m/z, (C) MS^2 scan in positive mode of precursor of 356.2923 m/z. (D) MS^2 scan in positive mode of dimer of 334.3102 m/z, (E) MS^2 scan in positive mode of dimer of 356.2923 m/z.

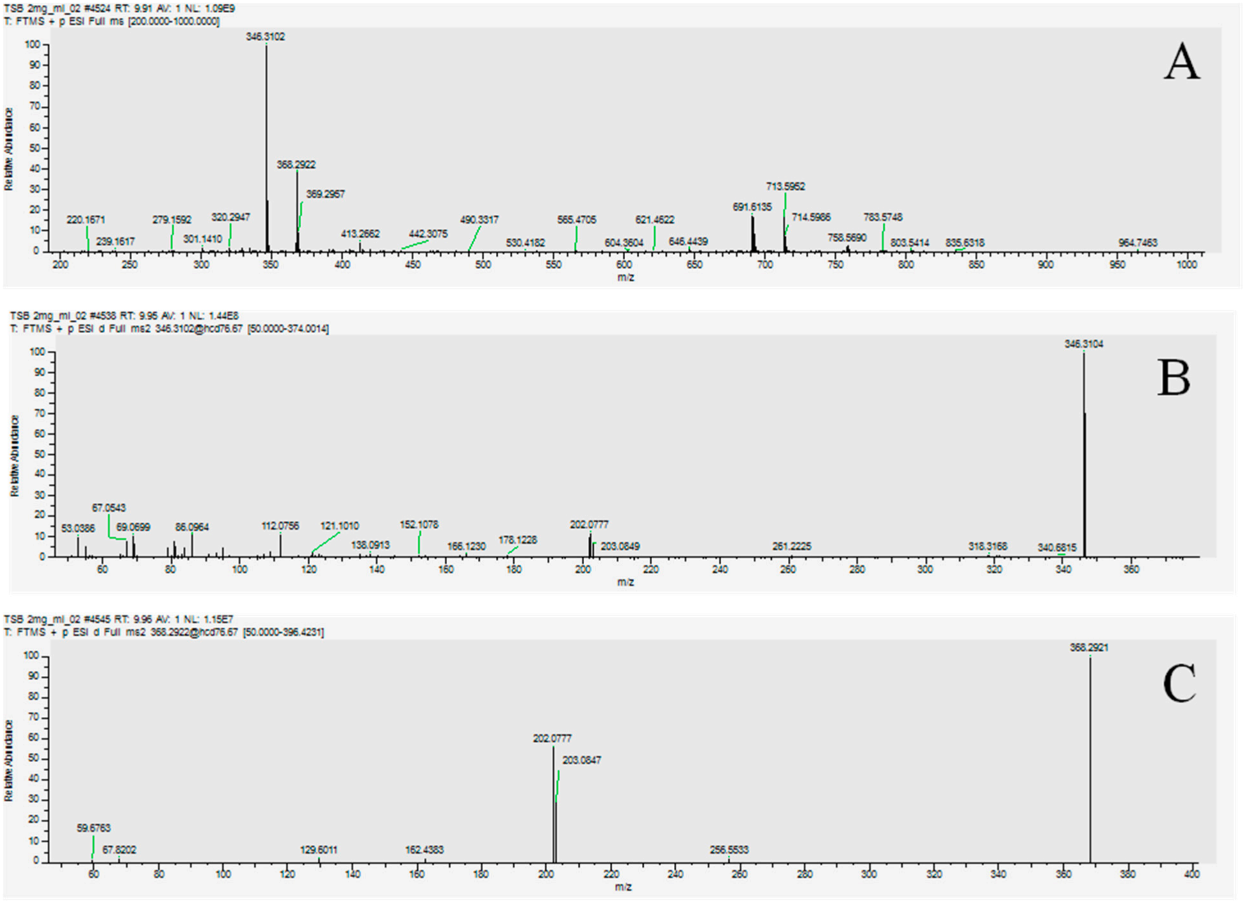

Figure S16 Mass spectra of 1-(piperidmyl)-2,4,12-octadecatrien-1-one (16) at retention time of 9.853 min. (A) full scan of 200-1000 m/z with positive mode, (B) MS^2 scan in positive mode of precursor of 346.3101 m/z, (C) MS^2 scan in positive mode of precursor of 368.2922 m/z.

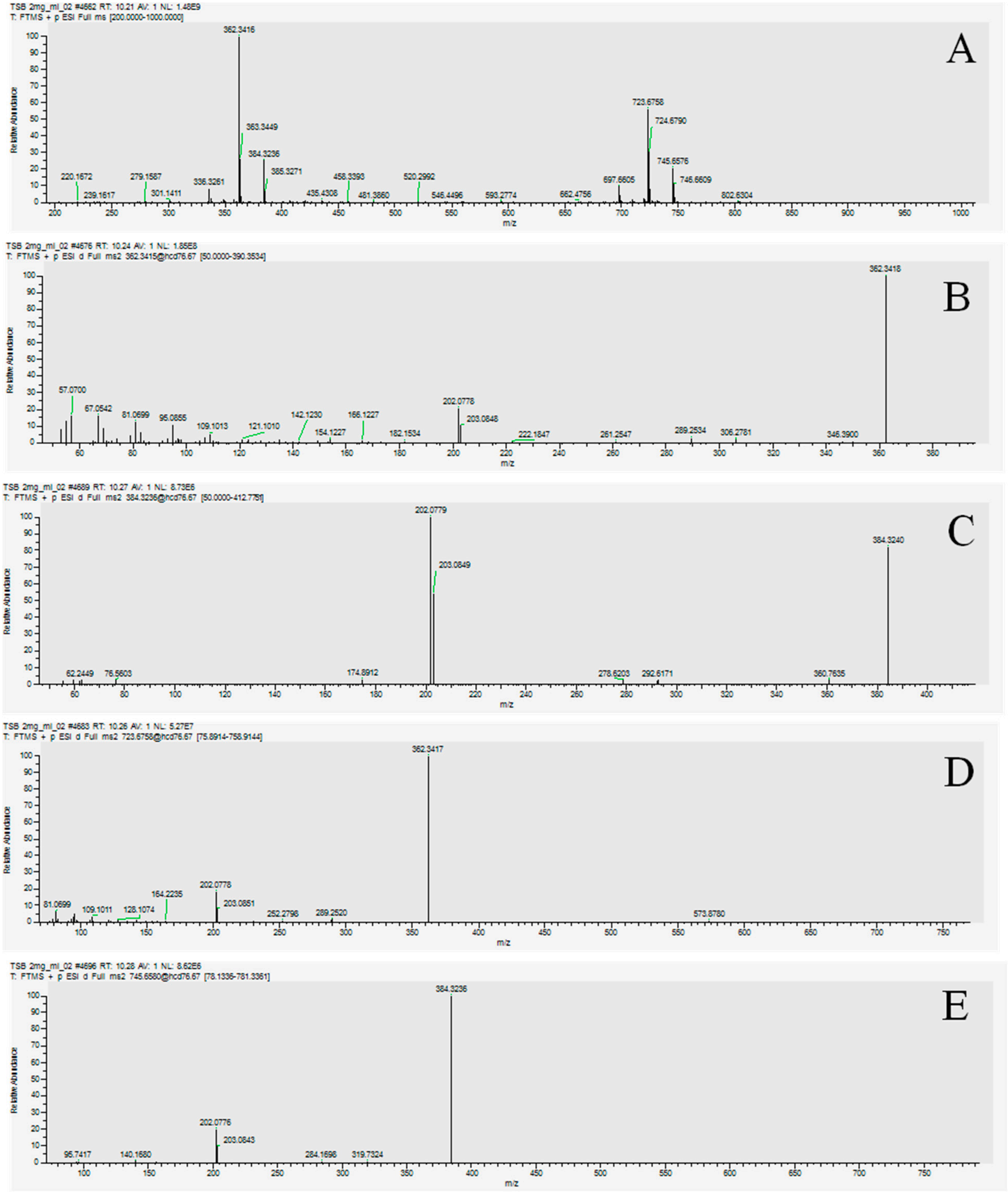

Figure S17 Mass spectra of N-isobutyl-2,4,14-eicosatrienamide (17) at retention time of 10.153 min. (A) full scan of 200-1000 m/z with positive mode, (B) MS² scan in positive mode of precursor of 362.3416 m/z, (C) MS² scan in positive mode of precursor of 384.3236 m/z. (D) MS² scan in positive mode of dimer of 362.3416 m/z, (E) MS² scan in positive mode of dimer of 384.3236 m/z.

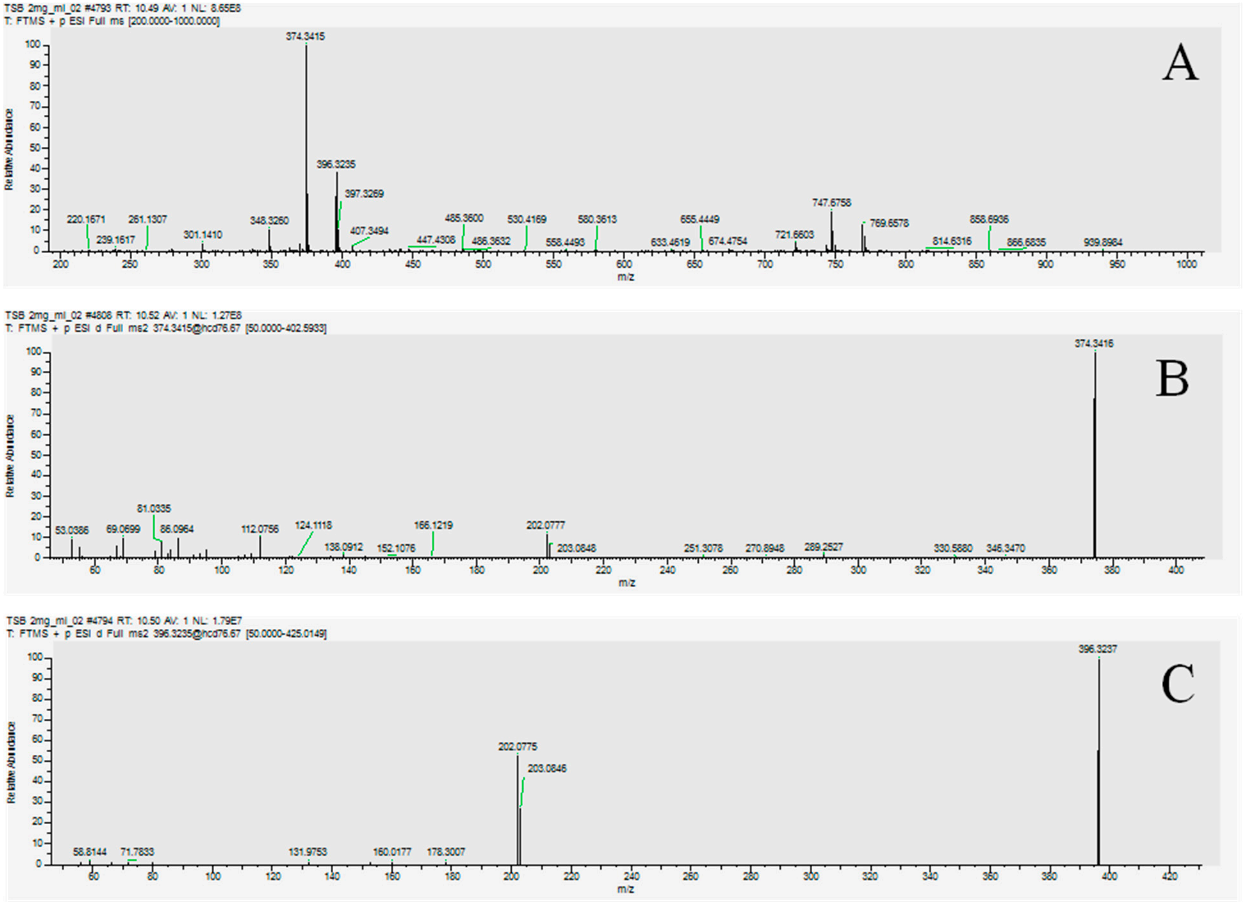

Figure S18 Mass spectra of 1-(piperidinyl)-2,4,14-eicosatrien-1-one (18) at retention time of 10.433min. (A) full scan of 200-1000 m/z with positive mode, (B) MS[2] scan in positive mode of precursor of 374.3415 m/z, (C) MS[2] scan in positive mode of precursor of 396.3235 m/z.

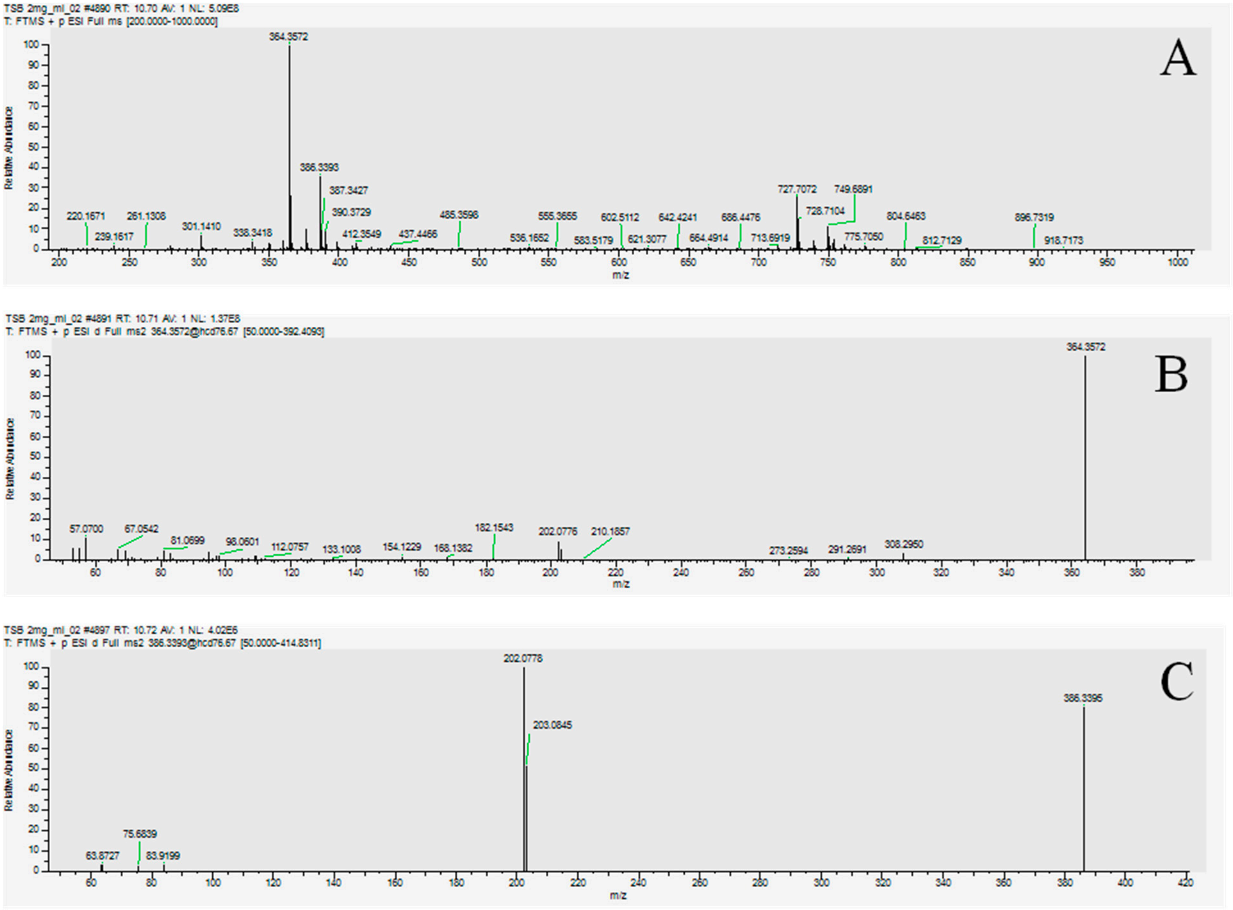

Figure S19 Mass spectra of N-isobutyl-2,4-eicosadienamide (19) at retention time of 10.650 min. (A) full scan of 200-1000 m/z with positive mode, (B) MS^2 scan in positive mode of precursor of 364.3572 m/z, (C) MS^2 scan in positive mode of precursor of 386.3392 m/z.

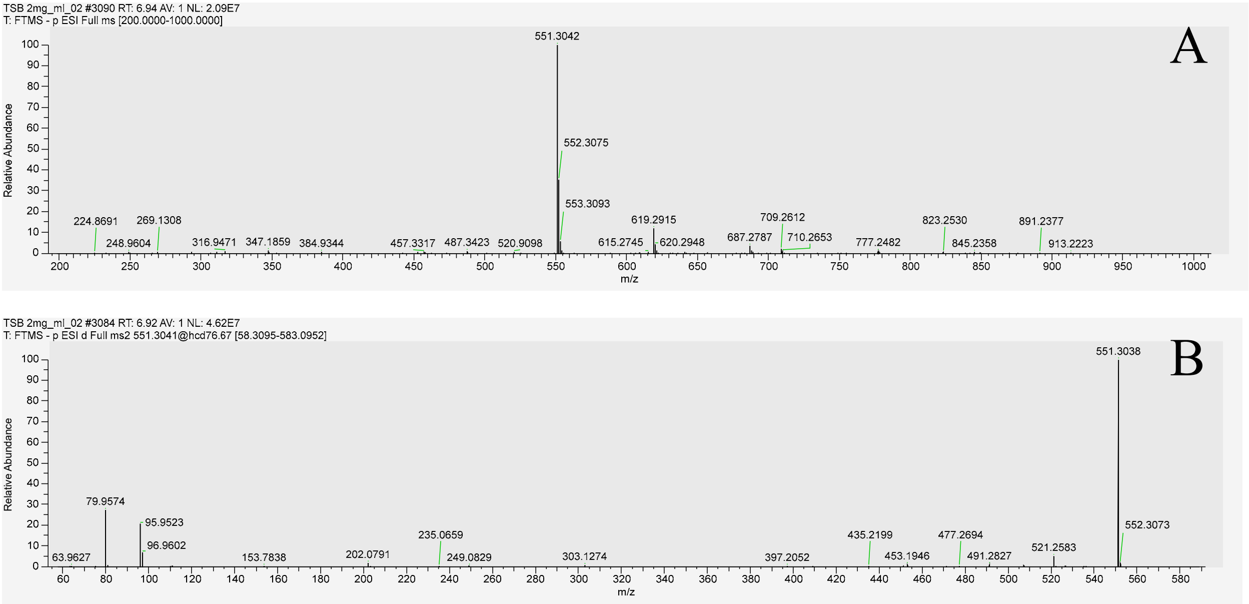

Figure S20 Mass spectra of unidentified compound (**20**) at retention time of 6.92 min. (A) full scan of 200-1000 m/z with negative mode, (B) MS² scan in negative mode of precursor of 551.3042 m/z.
